# Engineering the oleaginous yeast *Yarrowia lipolytica* to produce limonene from waste cooking oil

**DOI:** 10.1186/s13068-019-1580-y

**Published:** 2019-10-08

**Authors:** Yaru Pang, Yakun Zhao, Shenglong Li, Yu Zhao, Jian Li, Zhihui Hu, Cuiying Zhang, Dongguang Xiao, Aiqun Yu

**Affiliations:** State Key Laboratory of Food Nutrition and Safety, Key Laboratory of Industrial Fermentation Microbiology of the Ministry of Education, Tianjin Key Laboratory of Industrial Microbiology, Tianjin Engineering Research Center of Microbial Metabolism and Fermentation Process Control, College of Biotechnology, Tianjin University of Science and Technology, No.29 the 13th Street TEDA, Tianjin, 300457 People’s Republic of China

**Keywords:** *Yarrowia lipolytica*, Limonene, Mevalonate pathway, Metabolic engineering, Fermentation optimization, Waste cooking oil

## Abstract

**Background:**

Limonene is an important biologically active natural product widely used in the food, cosmetic, nutraceutical and pharmaceutical industries. However, the low abundance of limonene in plants renders their isolation from plant sources non-economically viable. Therefore, engineering microbes into microbial factories for producing limonene is fast becoming an attractive alternative approach that can overcome the aforementioned bottleneck to meet the needs of industries and make limonene production more sustainable and environmentally friendly.

**Results:**

In this proof-of-principle study, the oleaginous yeast *Yarrowia lipolytica* was successfully engineered to produce both d-limonene and l-limonene by introducing the heterologous d-limonene synthase from *Citrus limon* and l-limonene synthase from *Mentha spicata*, respectively. However, only 0.124 mg/L d-limonene and 0.126 mg/L l-limonene were produced. To improve the limonene production by the engineered yeast *Y. lipolytica* strain, ten genes involved in the mevalonate-dependent isoprenoid pathway were overexpressed individually to investigate their effects on limonene titer. Hydroxymethylglutaryl-CoA reductase (HMGR) was found to be the key rate-limiting enzyme in the mevalonate (MVA) pathway for the improving limonene synthesis in *Y. lipolytica*. Through the overexpression of *HMGR* gene, the titers of d-limonene and l-limonene were increased to 0.256 mg/L and 0.316 mg/L, respectively. Subsequently, the fermentation conditions were optimized to maximize limonene production by the engineered *Y. lipolytica* strains from glucose, and the final titers of d-limonene and l-limonene were improved to 2.369 mg/L and 2.471 mg/L, respectively. Furthermore, fed-batch fermentation of the engineered strains Po1g KdHR and Po1g KlHR was used to enhance limonene production in shake flasks and the titers achieved for d-limonene and l-limonene were 11.705 mg/L (0.443 mg/g) and 11.088 mg/L (0.385 mg/g), respectively. Finally, the potential of using waste cooking oil as a carbon source for limonene biosynthesis from the engineered *Y. lipolytica* strains was investigated. We showed that d-limonene and l-limonene were successfully produced at the respective titers of 2.514 mg/L and 2.723 mg/L under the optimal cultivation condition, where 70% of waste cooking oil was added as the carbon source, representing a 20-fold increase in limonene titer compared to that before strain and fermentation optimization.

**Conclusions:**

This study represents the first report on the development of a new and efficient process to convert waste cooking oil into d-limonene and l-limonene by exploiting metabolically engineered *Y. lipolytica* strains for fermentation. The results obtained in this study lay the foundation for more future applications of *Y. lipolytica* in converting waste cooking oil into various industrially valuable products.

## Background

Limonene, a monocyclic monoterpene, is an important biologically active natural product. It exists in two optical forms, i.e., d- and l-limonene with distinct properties and applications [1]. At present, limonene is widely used as a commercial flavor and fragrance in food, beverage, cosmetic and pharmaceutical industries because the d and l enantiomers possess pleasant lemon- or turpentine-like odor, respectively [2, 3]. Furthermore, d-limonene is used as a natural, environmentally friendly abluent in machinery, printing, aviation and electronic devices [4, 5], while l-limonene is a precursor for the biosynthesis of l-menthol, which is the major component of mint [6, 7]. In addition, limonene can also serve as a biofuel, a biopreservative, a versatile cleaning agent to replace toxic solvents and a key building block for a wide range of industrial products, including pharmaceuticals, nutraceuticals and other high-value fine chemicals [8–10]. Thus, there is an ever-increasing demand for limonene due to its vast range of applications. Currently, industrial production of limonene is mainly achieved by plant extraction, but the low abundance of limonene in plants renders their isolation from plant sources non-economically viable [11]. Likewise, chemical syntheses of limonene are limited by the complexity of the production equipment and low conversion rate of raw materials. These processes are also energy intensive and can cause environmental issues. Therefore, alternative production routes are urgently needed for industrial production of limonene. A promising approach is the engineering of microbes into microbial cell factories for de novo biosynthesis of limonene, and a variety of microbes have been engineered to produce d-limonene or l-limonene in many previous researches (Table 1).

**Table 1:** Some representative examples of limonene production in engineered microbial hosts

Host	Product	Titer	Yield	Productivity	Strategy	References
*E. coli*	l-Limonene	430 mg/L	–	5.97 mg/L/h	A plasmid with a truncated GPP synthase and limonene synthase gene was co-expressed with a plasmid increasing the MK and PMK levels (MBI-f operon) and replacing the *HMGS* and *HMGR* from *S. cerevisiae* (MevT operon) with those from *S. aureus* (MTSA operon) using different IPTG levels	[28]
	l-Limonene	2700 mg/L_org_	–	60 mg/L_org_/h	(1) Truncated *LS* gene and truncated *GPPS2* gene with all the genes of the MVA pathway, that convert endogenous acetyl-CoA into IPP and DMAPP from *S. cerevisiae* was co-expressed. (2) A two-liquid phase fed-batch fermentation with glycerol as carbon source were carried out in 3.1 L stirred-tank reactors	[35]
	l-Limonene	75.12 mg/L	–	3.13 mg/L/h	(1) Juice from sugar beets was utilized as a novel feedstock. (2) A synthetic DXP operon (*dxs*-*ipphp*-*gpps*) encoding three rate-limiting enzymes from the MEP pathway was co-expressed with limonene synthases gene	[75]
*S. cerevisiae*	d-Limonene and l-limonene	0.12 mg/L d-limonene and 0.49 mg/L l-limonene	–	1.67 µg/L/h d-limonene and 6.8 µg/L/h l–limonene	The truncated limonene synthases with Cytochrome P450 reductase (*CPR1*) (Gal1 promoter) were co-expressed, and a setup was built which allowed trapping of exhaust volatiles during growth	[33]
*Y. lipolytica*	d-Limonene and l-limonene	11.705 mg/L d-limonene and 11.088 mg/L l-limonene	0.443 mg/g DCW for d-limonene and 0.385 mg/g DCW for l-limonene	0.0542 mg/L/h d-limonene and 0.0513 mg/L/h l-limonene	(1) The heterologous d-limonene synthase from *Citrus limon* and l-limonene synthase from *Mentha spicata* with ten homologous genes involved in the MVA pathway were overexpressed, respectively. (2) Fermentation conditions of engineered strains were optimized. (3) Fed-batch fermentation of engineered strains was used. (4) WCO was used as carbon resource	This work
	d-Limonene	23.56 mg/L	1.36 mg/g DCW	0.33 mg/L/h	The truncated genes *tLS* and *tNDPS1* with *HMG1* and *ERG12* were co-expressed	[72]
*Anabaena* sp. PCC 7120	l-Limonene	–	–	172.7 µg/L/48 h	A synthetic DXP operon (*dxs*-*ipphp*-*gpps*) encoding three rate-limiting enzymes from the MEP pathway was co-expressed with limonene synthases gene	[76]
*Synechococcus* sp. PCC 7002	l-Limonene	4 mg/L	–	0.042 mg/L/h	The (−)-*4S*-limonene synthase gene was expressed	[30]
*Synechocystis* sp. PCC 6803	d-Limonene	0.564 mg/L	–	2.33 µg/L/h	Introducing an additional copy of three genes (*dxs*, *crtE* and *ipi*) under the control of a strong trc promoter	[77]

*Yarrowia lipolytica*, a non-conventional oleaginous yeast, is an attractive biochemical production host that can be employed for the production of food-grade products since it is classified by the US Food and Drug Administration as generally recognized as safe (GRAS) [12]. With the completion of the genome sequencing of *Y. lipolytica* and generation of available genetic tools for genetic manipulation, it has received increasing attention from researchers for use in metabolic engineering to produce valuable compounds. In recent years, *Y. lipolytica* has shown its versatility and importance as a production host by successfully demonstrating its utilization for a wide range for metabolic engineering and biotechnological applications [13–15].

**Fig. 1:**
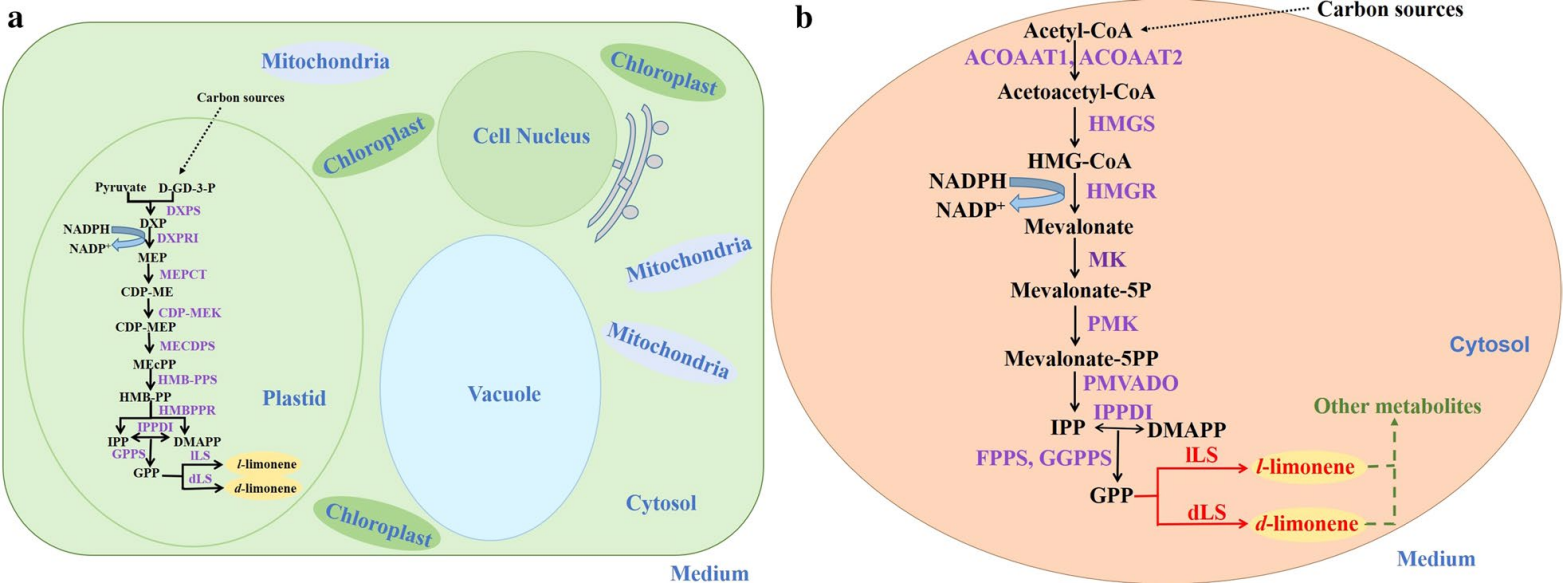
Biosynthesis pathway for limonene production in plants and the yeast *Y. lipolytica*. Limonene is biosynthesized from the precursor GPP by enzymatic biotransformation with d- or l-limonene synthetase. IPP and DMAPP are converted to GPP in both plant and *Y. lipolytica*. **a** Biosynthesis pathway for limonene production in plants. Plants produce GPP via the methylerythritol phosphate pathway from pyruvate and glyceraldehyde-3-phosphate. **b** Biosynthesis pathway for limonene production in *Y. lipolytica*. Yeast rely on the mevalonate pathway to produce GPP from acetyl-CoA. Since LS is not present in *Y. lipolytica* to construct a complete limonene pathway in *Y. lipolytica*, two heterologous genes encoding d-limonene synthase (*dLS*, from *C. limon*) and l-limonene synthase gene (*lLS*, from *M. spicata*) were introduced (shown in red). And downstream metabolic pathways of limonene may lie in yeast (shown in blue). The endogenous MVA pathway enzymes (purple) that were overexpressed in the engineered *Y. lipolytica* strains. Enzymes involved in the MEP pathway in plants and in the MVA pathway in *Y. lipolytica* are shown in parentheses. Homologous enzymes found in *Y. lipolytica* are shown in purple. *DXPS* DXP synthase, *DXPRI* DXP-reductoisomerase, *MEPCT* MEP cytidylyltransferase, *CDP-MEK* CDP-ME kinase, *MECDPS* MECDP-synthase, *HMBPPS* (E)-4-hydroxy-3-methylbut-2-enyl-diphosphate synthase, *HMBPPR* HMBPP reductase, *IPPDI* isopentenyl-diphosphate delta-isomerase, *GPPS* geranyl-diphosphate synthase, *lLS* l-limonene synthase, *dLS* d-limonene synthase, *ACOAAT1* acetyl-CoA C-acetyltransferase 1, *ACOAAT2* acetyl-CoA C-acetyltransferase 2, *HMGS* hydroxymethylglutaryl-CoA synthase, *HMGR* hydroxymethylglutaryl-CoA reductase, *MK* mevalonate kinase, *PMK* phosphomevalonate kinase, *PMVADO* diphosphomevalonate decarboxylase, *IPPDI* isopentenyl-diphosphate delta-isomerase, *GGPPS* geranylgeranyl diphosphate synthase, type III, *FPPS* farnesyl diphosphate synthase

In limonene-producing plants, limonene is biosynthesized from the precursor geranyl diphosphate (GPP) by enzymatic biotransformation with *d*- or l-limonene synthetase (Fig. 1a) [16]. While plants produce GPP via the methylerythritol phosphate (MEP) pathway from pyruvate and glyceraldehyde-3-phosphate (Fig. 1a) [17, 18], yeasts rely on the mevalonate (MVA) pathway to produce GPP from acetyl-CoA (Fig. 1b). Considering these requirements for implementing the limonene biosynthesis pathway, *Y. lipolytica* is an excellent microbial production host because (1) being an oleaginous yeast with an abundant pool of intracellular acetyl-CoA [13], it has great potential to be rewired to overproduce GPP to serve as a precursor for biotransformation to limonene and (2) being an eukaryote, it is suited for heterologous expression of the plant-derived d- and l-limonene synthases. Moreover, a unique characteristic of *Y. lipolytica* is that it can grow on a wide range of cheap hydrophobic and hydrophilic carbon sources, including alkanes, alkenes, fats, alcohols and organic acids [12, 19–24], thus it can be used as an effective whole-cell biocatalyst to bioremediate polluted environments [25]. Notably, this organism has been exploited to utilize waste cooking oils (WCO) as carbon sources for growth and substrates for bioconversion [26, 27]. Therefore, the biosynthesis of limonene from WCO with engineered *Y. lipolytica* can be a sustainable and economical avenue for production of the valuable and versatile chemical.

In this study, we demonstrated the engineering and optimization of *Y. lipolytica* toward limonene production from WCO. *Y. lipolytica* was first engineered to produce d-limonene and l-limonene by heterologous expression of selected d-limonene synthase gene and l-limonene synthase gene, respectively. Subsequently, ten genes involved in the MVA pathway were overexpressed to identify the key enzymes for improving the yields of d-limonene and l-limonene. To further enhance the yields of d-limonene and l-limonene, we varied the parameters for cultivating the engineered *Y. lipolytica* strains to optimize the fermentation conditions. Finally, the potential of using WCO as the carbon source for limonene production was investigated with the engineered *Y. lipolytica* strains. We envision that this work will provide insights in developing strategies for exploiting *Y. lipolytica* as a microbial cell factory for sustainable and economical production of valuable chemicals from waste oleaginous feedstock.

## Results and discussion

### Heterologous production of limonene in *Y. lipolytica* through limonene synthase expression

*Yarrowia lipolytica* Po1g KU70Δ was used as the platform strain for limonene production in this study. This is because, in addition to having relatively high level of intracellular acetyl-CoA as aforementioned, the tolerance of this oleaginous strain to d-limonene or l-limonene was found to be much higher than that of the conventional yeast host *Saccharomyces cerevisiae* (Additional file 1: Fig. S1). Furthermore, it was observed that the rate of precise homologous recombination in the Po1g KU70Δ strain is much higher than that of the parent strain Po1g, which will facilitate genomic manipulation.

**Fig. 2:**
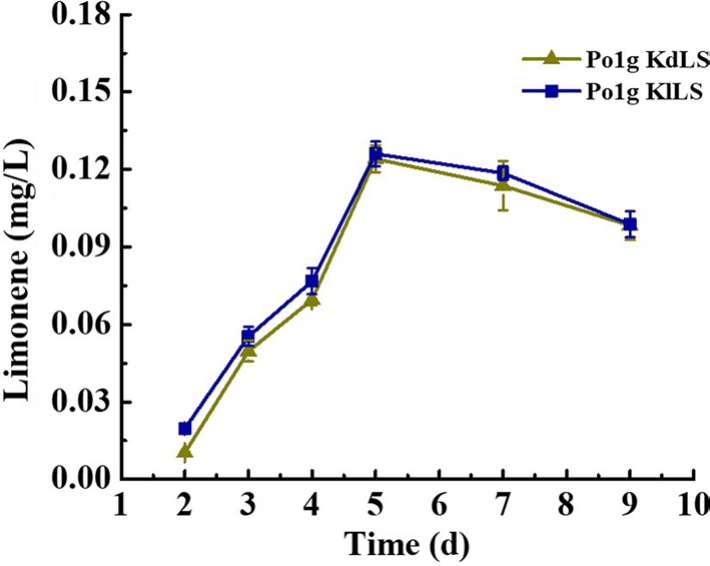
Time course of d-limonene and l-limonene production in the engineered *Y. lipolytica* strains. The cells of Po1g KdLS and Po1g KlLS were cultivated at 30 °C and 200 rpm with an initial OD_600_ of 0.1 in 30 mL of liquid YPD medium in a 250-mL shake flask, and limonene titers were determined at the 2-, 3-, 4-, 5-, 7- and 9-day time-points. To avoid loss of limonene during cultivation, 8% of *n*-dodecane overlay was added into the YPD medium prior to cultivation.  : production of d-limonene by Po1g KdLS.  : production of l-limonene by Po1g KlLS. Bars represent limonene titers. All values presented are the mean of three biological replicates ± standard deviation

As *Y. lipolytica* is not a natural producer of limonene, heterologous enzymes need to be expressed to convert the GPP synthesized to limonene via the MVA pathway. To this end, d-limonene synthase gene (*dLS*) from *Citrus limon* and l-limonene synthase gene (*lLS*) from *Mentha spicata* were selected for production of d-limonene and l-limonene, respectively, as *dLS* gene and *lLS* gene have been shown to be used successfully to produce d-limonene and l-limonene (Additional file 1: Fig. S2), respectively, in heterologous microbial hosts [28–32]. Subsequently, the codon-optimized *dLS* gene and *lLS* gene were individually integrated into the chromosome of *Y. lipolytica* Po1g KU70Δ strain and expressed under the strong constructive promoter hp4d. The resulting engineered strains Po1g KdLS and Po1g KlLS were cultured in the YPD medium. Overlaying with *n*-dodecane appears to be the most efficient method to recover the limonene production in *Y. lipolytica*, because this method has been proven very efficient for trapping large quantities of volatile substances accumulated in different microbes [30, 33–35], which may be exported by passive diffusion or active transporters [36, 37]. And it has no significant effects on the cell growth of *Y. lipolytica* (Additional file 1: Fig. S3). Thus, a 10% *n*-dodecane overlay was first added to the culture to capture the volatile limonene. The 9-day time courses of d-limonene and l-limonene production titers by Po1g KdLS and Po1g KlLS, respectively, are shown in Fig. 2. The titers of d-limonene and l-limonene increased continuously from the beginning of cultivation up to day 5, reaching a peak titer of 0.124 mg/L for d-limonene and 0.126 mg/L for l-limonene. Subsequently, the titers of d-limonene and l-limonene declined gradually. This is probably because of ceasing production of limonene upon glucose depletion, as d-limonene and l-limonene titers further increased upon supplementation with additional glucose (Additional file 1: Fig. S4). It was previously reported that carbon starvation brings about an almost complete loss of fermentative capacity [38]. Subsequently, P450 monooxygenases present in *Y. lipolytica* for alkane assimilation may have oxidized the exocyclic methyl group of limonene and divert the flux to other side-products [39–41]. In addition, glucose depletion probably could reduce the transportation of limonene from inside of the cell to the outside by affecting multidrug resistance pump activity [42]. Hence, these results indicated the functional expression of the codon-optimized *dLS* gene and *lLS* gene in *Y. lipolytica* and these strains will serve as limonene-producing base strains for further engineering and optimization.

### Overexpression of endogenous genes involved in the MVA pathway of *Y. lipolytica* to further improve limonene production

**Fig. 3:**
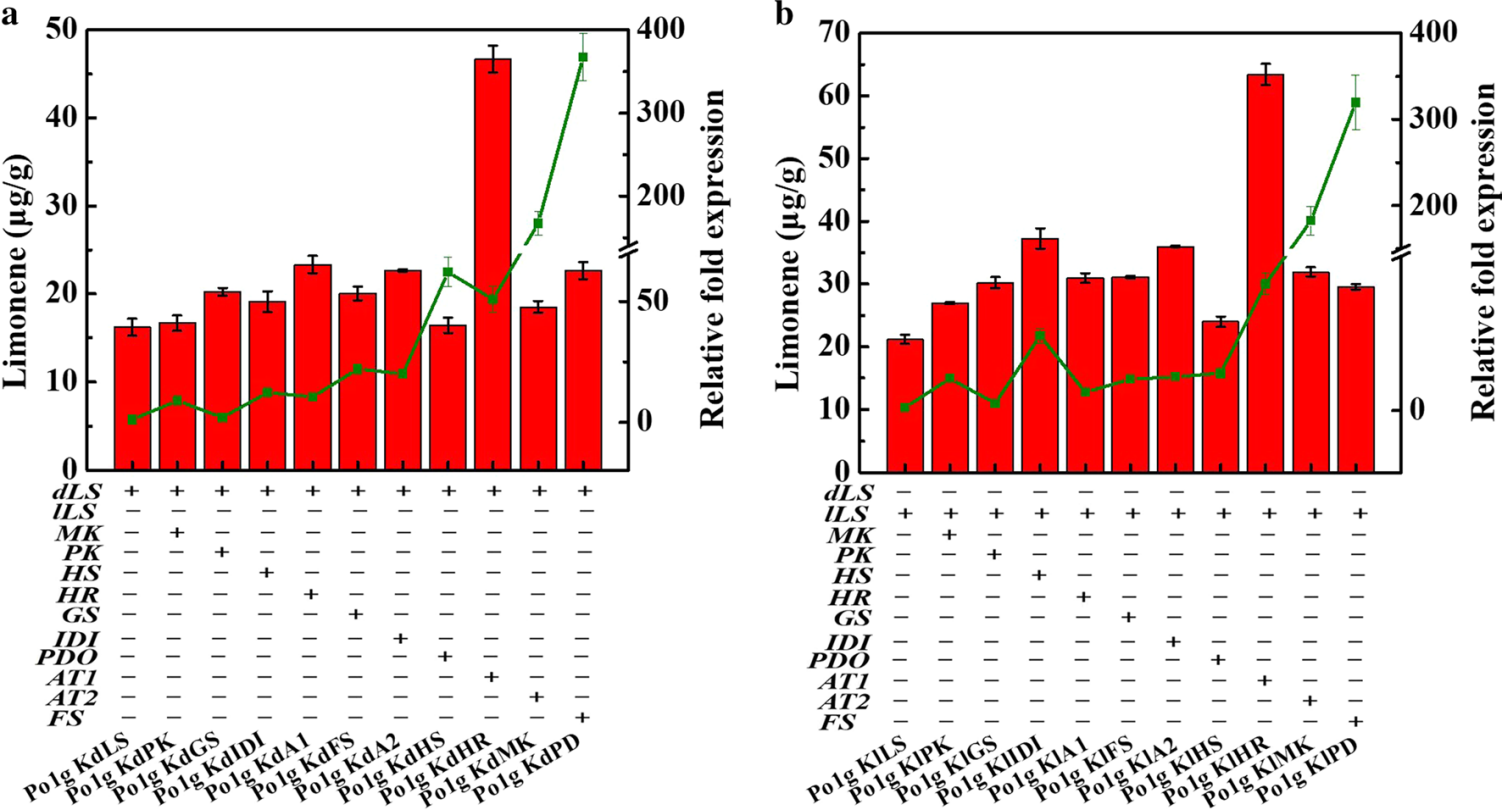
Effects of single-gene overexpression of genes involved in the MVA pathway on d-limonene or l-limonene production. **a** Effects of single-gene overexpression of genes involved in the MVA pathway on d-limonene production. Ten genes including *ACOAAT1*, *ACOAAT2*, *HMGS*, *HMGR*, *MK*, *PMK*, *PMVADO*, *IPPDI*, *GGPPS* and *FPPS* involved in MVA pathway were overexpressed individually with d-limonene synthase. **b** Effects of single-gene overexpression of genes involved in the MVA pathway on l-limonene production. Ten genes including *ACOAAT1*, *ACOAAT2*, *HMGS*, *HMGR*, *MK*, *PMK*, *PMVADO*, *IPPDI*, *GGPPS* and *FPPS* involved in MVA pathway were overexpressed individually with l-limonene synthase. Bars represent limonene yields and lines represent gene expression improvements over controls. Titers of d-limonene or l-limonene were quantified after 5 days of cultivation and the RNAs of ten genes were extracted after 3 days of cultivation in shake flasks with 30 mL of liquid YPD medium. Glucose was used as the carbon source. The *dLS*- or *lLS*-integrated strain cultivated in parallel was used as controls, and the gene encoding the β-actin protein was used as an internal standard. Relative gene expression measured relative to β-actin and normalized to controls was calculated as 2^−ΔΔ*C*T^. All values presented are the mean of three biological replicates ± standard deviation

To further enhance limonene production following the successful biosynthesis of limonene in *Y. lipolytica*, the genes involved in the MVA pathway were overexpressed in an attempt to increase the flux towards limonene. Ten genes, consisting of *ACOAAT1*, *ACOAAT2*, *HMGS*, *HMGR*, *MK*, *PMK*, *PMVADO*, *IPPDI*, *GGPPS* and *FPPS* (Fig. 1b), were overexpressed individually and investigated for their effects on limonene overproduction to determine the key genes that are critical for limonene biosynthesis in the MVA pathway. To this end, twenty strains overexpressing the codon-optimized *dLS* or *lLS* gene with one of the ten endogenous genes in the MVA pathway of *Y. lipolytica* were constructed; all the genes were integrated into the chromosome of *Y. lipolytica* Po1g KU70Δ and expressed with the strong constitutive promoter hp4d. The transcription levels of the genes were determined by qRT-PCR and verified to have increased, suggesting that all the ten individually integrated genes were overexpressed (Fig. 3). The twenty engineered strains were cultured in YPD medium for 5 days in shake flasks. Individual overexpression of the selected genes did not cause any adverse effect on cell growth (Additional file 1: Fig. S5). The limonene titers of the strains showed that the overexpression of the individual corresponding genes could improve the limonene production compared to control strains expressing only the respective limonene synthases (Fig. 3). The HMGR-overexpressed strains, Po1g KdHR (Fig. 3a) and Po1g KlHR (Fig. 3b), achieved the highest titers of 46.703 μg/g DCW (0.286 mg/L) and 63.418 μg/g DCW (0.367 mg/L) for d-limonene and l-limonene after 5 days of cultivation, respectively. These represent a 288% increase in d-limonene titer over the Po1g KdLS strain and a 299% increase in l-limonene titer over the Po1g KlLS strain. However, the titer change in limonene was not consistent with the change in the levels of gene transcription, suggesting that the highest limonene titer, which was achieved by the overexpression of HMGR, was not attributed to the highest increase in gene expression level (Fig. 3). Our results indicate that the 3-hydroxy-3-methylglutaryl-coenzyme A (HMG-CoA) reductase of *Y. lipolytica* encoded by *HMGR* is the key regulatory step controlling isoprenoid metabolism, which improved limonene synthesis in *Y. lipolytica* upon overexpression. In numerous studies, HMG-CoA reductase has been proven to be a rate-limiting step in the production of the wide variety of molecules derived from mevalonate by the mevalonate pathway [43–47]. Thus, the strains Po1g KdHR and Po1g KlHR were chosen for further enhancement in limonene production by fermentation optimization.

### Fermentation optimization of the engineered *Y. lipolytica* strains

In microorganisms, the biosynthesis of most metabolites is strongly affected by fermentation parameters such as temperature, pH, rotation speed, initial cell density, additives and nutrients. We, therefore, hypothesized that the capability of our engineered *Y. lipolytica* strains in producing limonene could be further improved by optimizing the fermentation conditions. To our knowledge, this is the first report on the optimization of fermentation conditions for limonene production with microbes.

#### Effect of temperature on the accumulation of limonene produced by the engineered *Y. lipolytica* strains

**Fig. 4:**
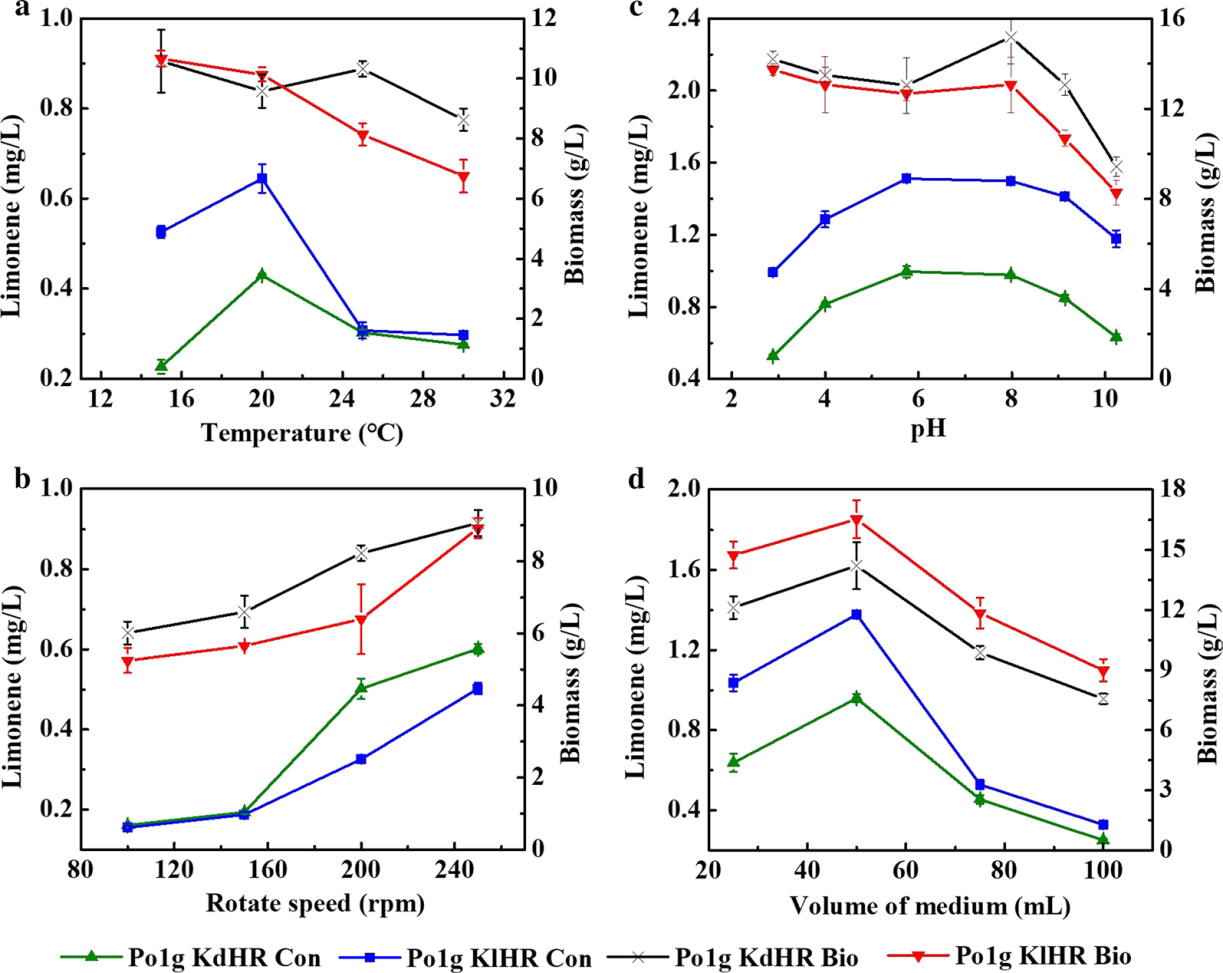
Effect of temperature, initial pH, rotation speed and volume of medium on d-limonene or l-limonene accumulation of Po1g KdHR or Po1g KlHR. **a** Effect of temperature on d-limonene or l-limonene accumulation of Po1g KdHR or Po1g KlHR. The cultivation was performed at 200 rpm with an initial OD_600_ of 0.1 and 8% of *n*-dodecane in 30 mL of liquid YPD medium in a 250-mL shake flask for 5 days at different temperatures (15, 20, 25 and 30 °C). **b** Effect of rotation speed on d-limonene or l-limonene accumulation of Po1g KdHR or Po1g KlHR. The cells were cultivated at 30 °C with an initial OD_600_ of 0.1 and 8% of *n*-dodecane in 30 mL of liquid YPD medium in a 250-mL shake flask for 5 days at different rotation speeds (100 rpm, 150 rpm, 200 rpm and 250 rpm). **c** Effect of initial pH on d-limonene or l-limonene accumulation of Po1g KdHR or Po1g KlHR. The cultivation was performed at the optimum fermentation temperature of 20 °C and the optimum rotation speed of 250 rpm, with an initial OD_600_ of 0.1 and 8% of *n*-dodecane in 30 mL of liquid YPD medium in a 250-mL shake flask for 5 days at six different initial pH values ranging from 3 to 10. **d** Effect of volume of medium on d-limonene or l-limonene accumulation of Po1g KdHR or Po1g KlHR. The cultivation was performed at the optimum fermentation temperature of 20 °C, the optimum rotation speed of 250 rpm, with an initial OD_600_ of 0.1 and 8% of *n*-dodecane in a 250-mL shake flask for 5 days at different volumes of liquid YPD medium (25 mL, 50 mL, 75 mL and 100 mL).  : d-limonene accumulation of Po1g KdHR;  : l-limonene accumulation of Po1g KlHR;  : biomass accumulation of Po1g KdHR;  : biomass accumulation of Po1g KlHR. All values presented are the mean of three biological replicates ± standard deviation

Temperature, one of the important environmental factors for gene expression and enzyme activity that are associated with microbial cell growth and metabolism, is often one of the key parameters affecting microbial fermentation performance [48, 49]. To investigate the effect of cultivation temperature on the accumulation of d-limonene and l-limonene during glucose fermentation by the engineered Po1g KdHR and Po1g KlHR strains, the cells were cultivated at 200 rpm with an initial OD_600_ of 0.1 and 8% of *n*-dodecane in 30 mL of liquid YPD medium in a 250-mL shake flask for 5 days at various cultivation temperatures, i.e., 15, 20, 25, and 30 °C. As shown in Fig. 4a, while there was a slight decrease in the biomass of Po1g KdHR and Po1g KlHR when the cultivation temperature was increased from 15 to 20 °C, the titers of d-limonene and l-limonene increased sharply. However, increasing the cultivation temperature from 20 to 30 °C drastically reduced the titers for both strains. Therefore, after 5 days of cultivation, the maximum limonene titers (0.427 mg/L for d-limonene and 0.632 mg/L for l-limonene) were achieved from the Po1g KdHR and Po1g KlHR strains, respectively, at 20 °C, suggesting that this is the optimum temperature for the production of d-limonene and l-limonene from the engineered *Y. lipolytica* strains. It is well known that low temperature is beneficial for decreasing the evaporation of volatile substances, and it was also demonstrated that the evaporation of limonene was decreased at low temperature (Additional file 1: Fig. S6). Therefore, it is 20 °C not 15 °C nor 30 °C that improved limonene production, which can probably be attributed to the combined effect of increased fermentation yield of limonene and the suppression of evaporation.

#### Effect of rotation speed on the accumulation of limonene produced by the engineered *Y. lipolytica* strains

Rotation speed plays a very important role in aerobic fermentation because it influences the mass transfer of gases, such as O_2_ and CO_2_, in and out of the growth medium [50]. Therefore, the effect of rotation speed on the accumulation of d-limonene and l-limonene by the engineered Po1g KdHR and Po1g KlHR strains was herein investigated. To this end, the cultivation was performed at 30 °C with an initial OD_600_ of 0.1 and 8% of *n*-dodecane in 30 mL of liquid YPD medium in a 250-mL shake flask for 5 days while four different rotation speeds (100, 150, 200, and 250 rpm) were applied to the cultures. As shown in Fig. 4b, the biomass gradually increased and limonene production progressively improved as the rotation speed increased from 100 to 250 rpm, at which the maximum titers of limonene (0.603 mg/L for d-limonene and 0.509 mg/L for l-limonene) were achieved from Po1g KdHR and Po1g KlHR. Therefore, these results imply that the improved titers of limonene may be attributed to the increased biomass. However, the effect of rotation speed over 250 rpm was not tested because of the speed limit of the routine incubator shaker used in this study. Thus, the optimum rotation speed for production of d-limonene and l-limonene from the engineered *Y. lipolytica* strains was established to be 250 rpm. These results suggest that high rotation speed in shake flask cultures is imperative to ensure sufficient oxygen supply for the optimum production of limonene by the strictly aerobic yeast *Y. lipolytica* [51].

#### Effect of pH on the accumulation of limonene produced by the engineered *Y. lipolytica* strains

Extracellular pH is often a critical parameter to obtain optimal microbial activity in the culture media. In particular, pH changes directly influence membrane potential, proton motive force and consequently substrate utilization and product profile [49]. In this regard, the pH change in the medium during time of the culture was measured. We found that the pH of the fermentation medium did not significantly change at the initial stage of the *Y. lipolytica* cultivation (from day 1 to day 3, Additional file 1: Fig. S7), although the pH rose to around 8.5 at the end of the cultivation. Therefore, we propose initial pH could be a limiting factor for limonene production in the process of fermentation by engineered *Y. lipolytica* strains. To verify this hypothesis, six different initial pH values ranging from 3 to 10 were used to determine the effect of initial pH value of the YPD medium on the accumulation of d-limonene and l-limonene by the engineered *Y. lipolytica* Po1g KdHR and Po1g KlHR strains. The cultivation was performed at the optimum fermentation temperature of 20 °C and the optimum rotation speed of 250 rpm, with an initial OD_600_ of 0.1 and 8% of *n*-dodecane in 30 mL of liquid YPD medium in a 250-mL shake flask for 5 days. Limonene production by the engineered *Y. lipolytica* strains was possible over a wide range of pH values but the titers varied. As shown in Fig. 4c, when the pH was raised from 2.88 to 5.74, there was a slight decrease in biomass as the titers of d-limonene and l-limonene increased gradually to peak at pH 5.74. Raising the pH from 5.74 and 7.98 increased the biomass but the titers of both d-limonene and l-limonene were almost constant. Further increasing the pH from 7.98 to 10.24 reduced both the biomass and the titers. In general, the reason why the highest titers of both d-limonene and l-limonene were obtained at pH 5.74–7.98 is that these pHs are close to the physiological pH and a large deviation of the extracellular environment from the physiological pH can lead to changes in cellular gene expression, enzyme activity, signaling pathways and ultimately the biological activity of microorganisms. Since the highest limonene titers were achieved at pH 5.74, this pH was established to be optimum pH for production of d-limonene and l-limonene from the engineered *Y. lipolytica* strains.

#### Effect of the volume of culture medium on the accumulation of limonene produced by the engineered *Y. lipolytica* strains

As mentioned above, the oleaginous yeast *Y. lipolytica* is a strictly aerobic yeast. During the cultivation process, different levels of dissolved oxygen in the medium can greatly influence cell growth of this yeast, and thus production of target products. Similar to rotation speed, the volume of liquid culture medium is another factor that can affect the level of dissolved oxygen in the medium. In general, the level of dissolved oxygen in the medium would be high when the volume of culture medium is low. However, low culture volume could also lead to insufficient supply of nutrient and energy. To study the effect of the volume of liquid YPD medium on the accumulation of d-limonene and l-limonene, the engineered *Y. lipolytica* strains were cultivated in different volumes of liquid YPD medium (25 mL, 50 mL, 75 mL, and 100 mL) at the optimum fermentation temperature of 20 °C, the optimum rotation speed of 250 rpm, with an initial OD_600_ of 0.1 and 8% of *n*-dodecane in a 250-mL shake flask for 5 days. As shown in Fig. 4d, the biomass and the titers of both d-limonene and l-limonene were enhanced sharply when the culture volume was increased from 25 to 50 mL but further increase in culture volume to 75 and 100 mL caused drastic decrease in the biomass and titers. The maximum titers of limonene (1.045 mg/L for d-limonene and 1.530 mg/L for l-limonene) were achieved at the peak of biomass from 50 mL of YPD medium, hence implying that the improved titers can be attributed to the increased biomass. These results also indicate that 50 mL of YPD medium is the optimum culture volume for both availability of dissolved oxygen and supply of nutrients in 250-mL shake flasks for the production of d-limonene and l-limonene from the engineered *Y. lipolytica* strains. To further investigate the effects of different levels of dissolved oxygen in the medium on the accumulation of d-limonene and l-limonene during glucose fermentation by the engineered Po1g KdHR and Po1g KlHR strains, the fermentation was carried out in a 5-L laboratory fermenter. However, the dissolved oxygen level was unstable during the batch fermentation of the engineered *Y. lipolytica* strains (Additional file 1: Fig. S8). One possible reason is that the oxygen consumption by the yeast cells was too fast for the effects of culture volume and rotational speed to be stable. In addition, it is possible that inappropriate types and volumes of antifoaming agents added caused the phenomenon. The effect of different dissolved oxygen level on the accumulation of limonene was thus not determined by this experiment in fermenter.

#### Effect of the volume of *n*-dodecane on the accumulation of limonene produced by the engineered *Y. lipolytica* strains

**Fig. 5:**
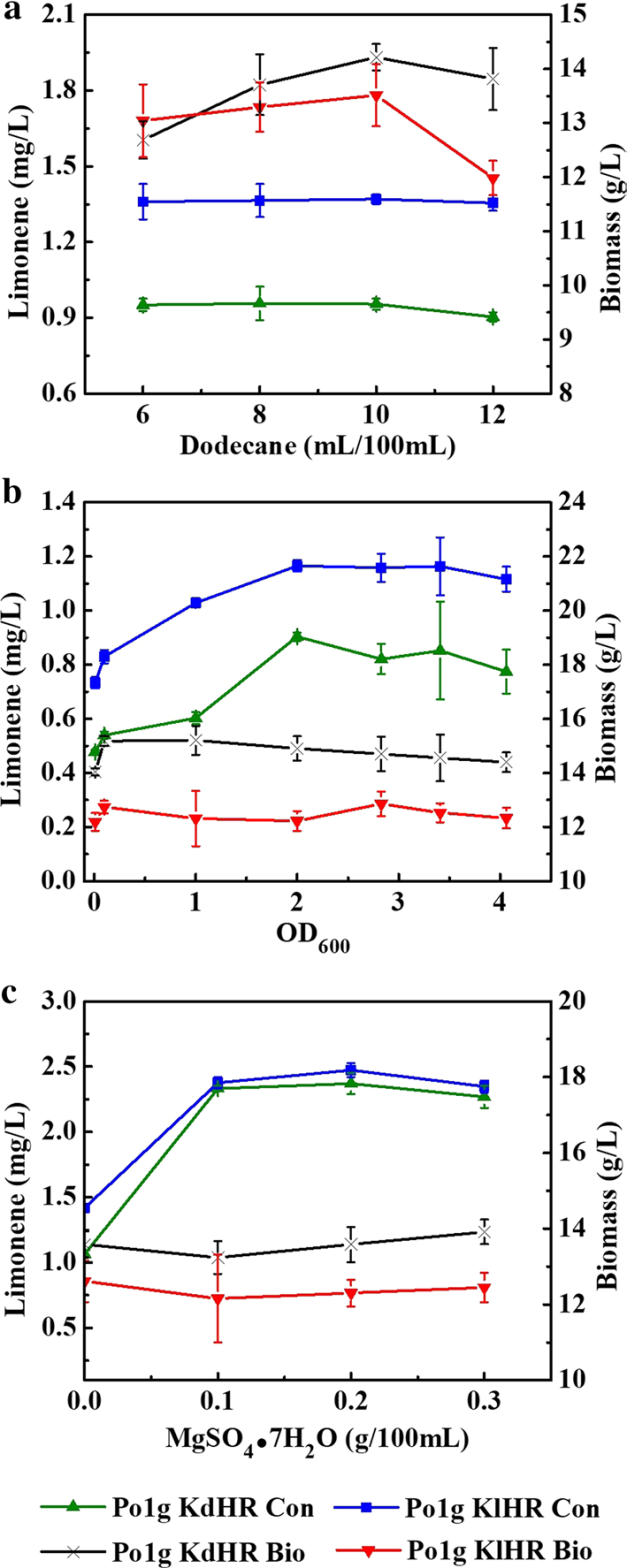
Effect of volume of *n*-dodecane, initial OD_600_ and MgSO_4_·7H_2_O on d-limonene or l-limonene accumulation in Po1g KdHR or Po1g KlHR. **a** Effect of volume of *n*-dodecane on d-limonene or l-limonene accumulation in Po1g KdHR or Po1g KlHR. The cells were cultivated at the optimum fermentation temperature of 20 °C, the optimum rotation speed of 250 rpm, with an initial OD_600_ of 0.1 in 30 mL of liquid YPD medium in a 250-mL shake flask for 5 days with different volumes of *n*-dodecane (6%, 8%, 10%, and 12%). **b** Effect of initial OD_600_ on d-limonene or l-limonene accumulation in Po1g KdHR or Po1g KlHR. The cells were cultivated at the optimum fermentation temperature of 20 °C, the optimum rotation speed of 250 rpm, with an initial OD_600_ of 0.1 and 8% of *n*-dodecane in 30 mL of liquid YPD medium in a 250-mL shake flask for 5 days with different initial OD_600_ (0.01, 0.1, 1.0 and 2.0). **c** Effect of volume of MgSO_4_·7H_2_O on d-limonene or l-limonene accumulation in Po1g KdHR or Po1g KlHR. The cultivation was performed at the optimum fermentation temperature of 20 °C, the optimum rotation speed of 250 rpm, the optimum initial OD_600_ of 2.0, the optimum pH of 5.74 and the best *n*-dodecane volume 10% in the optimal 50 mL-final-volume of medium in a 250-mL shake flask for 5 days with different concentrations of MgSO_4_·7H_2_O (0, 0.1%, 0.2%, and 0.3%).  : d-limonene accumulation of Po1g KdHR;  : l-limonene accumulation of Po1g KlHR;  : biomass accumulation of Po1g KdHR;  : biomass accumulation of Po1g KlHR. All values presented are the mean of three biological replicates ± standard deviation

Limonene, a highly volatile monoterpene, can be relatively easy to be captured by overlaying the microbial fermentation system with *n*-dodecane [30, 34, 35]. Although *n*-dodecane is a known carbon source for *Y. lipolytica*, it did not contribute significantly to the growth of our engineered strains in the rich medium that we used for cultivation (Additional file 1: Fig. S3). However, the optimum volume of *n*-dodecane overlay for efficient capturing of the limonene products from the fermentation broth of the engineered *Y. lipolytica* has not been determined. In this study, we investigated the efficiency of different *n*-dodecane overlay volume (6%, 8%, 10% and 12%) in capturing the limonene from the fermentation broth of Po1g KdHR and Po1g KlHR. The cells were cultivated at the optimum fermentation temperature of 20 °C and the optimum rotation speed of 250 rpm, with an initial OD_600_ of 0.1 in 30 mL of liquid YPD medium in a 250-mL shake flask for 5 days. As shown in Fig. 5a, increasing the volume of *n*-dodecane from 6 to 10% only led to a marginal improvement in the titers of d-limonene and l-limonene; further increase in *n*-dodecane volume to 12% decreased the limonene titer slightly. Hence, these results demonstrated that an excess of *n*-dodecane could not improve the capturing efficiency of the produced limonene. Since the highest titers of d-limonene and l-limonene were achieved with 10% *n*-dodecane, this was chosen as the optimum overlay volume for capturing the limonene produced from the engineered *Y. lipolytica* strains.

#### Effect of initial OD_600_ on the accumulation of limonene produced by the engineered *Y. lipolytica* strains

In many previous reports, using a relatively high initial OD_600_ is a successful strategy to reduce the fermentation time and overcome the inhibition by inhibitors present in the microbial fermentation system. However, the higher initial OD_600_ could result in the problems such as decreased dissolved oxygen level and increased by-product level in the microbial fermentation system [49, 52–55]. Therefore, the effect of initial OD_600_ on the accumulation of d-limonene and l-limonene by the engineered *Y. lipolytica* strains was herein investigated. The Po1g KdHR and Po1g KlHR strains were inoculated at different initial OD_600_ (0.01, 0.1, 1.0, and 2.0) and cultivated at the optimum fermentation temperature of 20 °C and the optimum rotation speed of 250 rpm, with 8% of *n*-dodecane in 30 mL of liquid YPD medium in a 250-mL shake flask for 5 days. As shown in Fig. 5b, the titers of both d-limonene and l-limonene were improved significantly when the initial OD_600_ was increased from 0.01 to 2.0, although the increase was more drastic in the range of 0.01–0.1 and more modest from 0.1 to 2.0. The titers of both d-limonene and l-limonene were not varied significantly when the initial OD_600_ was increased further from 2.0 to 4.06. In contrast, the biomass in general was not significantly varied. However, the fermentation time was not reduced and the biomass was not significantly varied with the increase of initial cell concentration. The maximum titer of limonene (0.704 mg/L for d-limonene and 1.265 mg/L for l-limonene) was obtained from Po1g KdHR and Po1g KlHR when the initial OD_600_ was 2.0, suggesting that this was the optimum initial OD_600_ for the production of d-limonene and l-limonene from the engineered *Y. lipolytica* strains.

#### Effect of Mg^2+^ concentration on the accumulation of limonene produced by the engineered *Y. lipolytica* strains

Essential trace elements and metallic salts are necessary for growth and metabolism of microorganisms, thus have effects on the intracellular accumulation of metabolites in microorganisms [56, 57]. Previously, it was proven that the addition of Mg^2+^ has great influence on fermentation performance of oleaginous microorganisms [58]. We thus hypothesized that the addition of Mg^2+^ will further enhance limonene production by the engineered *Y. lipolytica* strains. To verify this hypothesis, the culture medium was supplemented with different concentrations of MgSO_4_·7H_2_O (0%, 0.1%, 0.2%, and 0.3%). First, the effect of Mg^2+^ on growth of *Y. lipolytica* was investigated, and the results suggested that the effect of exogenous magnesium on the growth of the engineered *Y. lipolytica* strains was not significant under our experimental conditions (Additional file 1: Fig. S9). Then, Po1g KdHR and Po1g KlHR were cultivated in 250-mL shake flasks for 5 days with the optimum fermentation parameters determined afore, i.e., 50 mL of liquid YPD medium, temperature of 20 °C, rotation speed of 250 rpm, initial OD_600_ of 2.0, initial pH of 5.74 and 10% *n*-dodecane overlay. The results showed that the biomass was not significantly varied and the titers of d-limonene and l-limonene in the medium supplemented with Mg^2+^ were much higher than those without added Mg^2+^ (Fig. 5c). The maximum titers of limonene (2.268 mg/L for d-limonene and 2.347 mg/L for l-limonene) were achieved by Po1g KdHR and Po1g KlHR, respectively, with a supplement of additional 0.2% of MgSO_4_·7H_2_O, indicating that this is the optimum Mg^2+^ concentration for the production of d-limonene and l-limonene from the engineered *Y. lipolytica* strains. These results obtained could possibly be due to the fact that Mg^2+^ could enhance the activities of enzymes in the MVA pathway or limonene synthases in the engineered *Y. lipolytica* strains, which has been reported in the literature [29, 59]. Mn^2+^ was also considered as a factor that may affect the activity of enzymes in the MVA pathway or limonene synthases as described in some previous reports [31, 60]. Therefore, the effect of Mn^2+^ on the accumulation of limonene produced by the engineered *Y. lipolytica* strains was also investigated. The results suggest that the addition of Mn^2+^ has no significant effect on the enhancement of limonene production and even decreased limonene titer at high concentrations of Mn^2+^ (Additional file 1: Fig. S10). These results suggest that it is Mg^2+^ and not Mn^2+^ that improved the production of d-limonene and l-limonene more in this work. In addition, the carbon/nitrogen ratio was investigated by changing the concentration of peptone, the results suggested that the optimum peptone concentration was 2% (20 g/L), and this optimum peptone concentration was the concentration that we have used for the all the experiments (Additional file 1: Fig. S11).

**Fig. 6:**
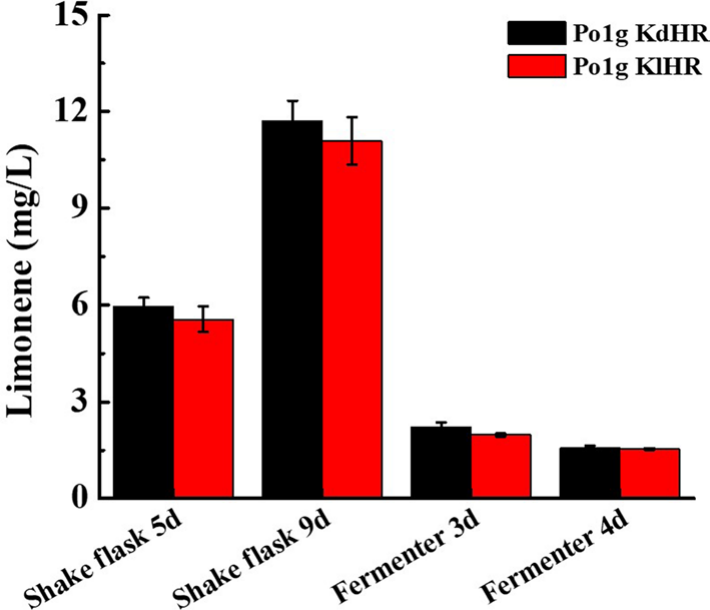
d-Limonene or l-limonene accumulation in Po1g KdHR or Po1g KlHR during fed-batch cultures on glucose. Fed-batch shake flask cultivation was carried out at 20 °C, 250 rpm. The fermentation condition of fed-batch fermentation in the fermenter was performed at 28 °C, pH 5.7 and with 20% dissolved oxygen. 50% glucose solution was continuously fed into the shake flask and fermenter (1 mL at every 24-h time-point and 120 mL over every 24-h cultivation period, respectively). All values presented are the mean of three biological replicates ± standard deviation

Subsequently, fed-batch fermentation of the engineered strains Po1g KdHR and Po1g KlHR was used to enhance limonene production in shake flasks and a 5 l-fermenter. After 9 days of cultivation in fed-batch fermentation of the engineered strains Po1g KdHR and Po1g KlHR in shake flasks, the limonene titers were determined and adjusted to the initial culture volume to account for the loss of growth medium and *n*-dodecane from evaporation; the remaining volume of culture was 43 mL, the remaining volume of *n*-dodecane was 4.2 mL, and pH of the cell culture was 7.8. As shown in Fig. 6, the titers of both d-limonene and l-limonene improved significantly upon fed-batch fermentation of the engineered strains Po1g KdHR and Po1g KlHR. The titers of d-limonene and l-limonene further increased when the fermentation time was lengthened to 9 days in shake flasks, leading to d-limonene and l-limonene titers of 11.705 mg/L (0.443 mg/g) and 11.088 mg/L (0.385 mg/g), respectively (the productivities of d-limonene and l-limonene were 0.0542 mg/L/h and 0.0513 mg/L/h, respectively). These enhanced titers represent over 3.5-fold improvement compared with the titers of 2.369 mg/L d-limonene and 2.471 mg/L l-limonene before using fed-batch fermentation. Fed-batch fermentation at a constant glucose feed rate was carried out in a 5-L fermenter, as it is an efficient measure to control oxygen consumption and ensure favorable cell growth [61]. The data of limonene fed-batch fermentation parameters in the 5-L fermenter are shown in Additional file 1: Fig. S12. However, the titers of both d-limonene and l-limonene declined markedly when fed-batch fermentation of the engineered strains Po1g KdHR and Po1g KlHR was carried out for 3 days in a 5 L-fermenter. The titers of d-limonene and l-limonene were only 2.222 mg/L and 1.975 mg/L, respectively (the productivities of d-limonene and l-limonene were 0.0309 mg/L/h and 0.0274 mg/L/h, respectively), and the titers of d-limonene and l-limonene further declined with increasing fermentation time. The low titers can possibly be attributed to non-optimal fed-batch medium and cultivation parameters (e.g., concentration of ions, pH and dissolved oxygen level) during the fed-batch fermentation of the engineered *Y. lipolytica* strains. Importantly, *n*-dodecane overlay for capturing volatile limonene, which played a critical role for improving limonene production in shake flasks, might not be beneficial in the fermenter. Particularly, the sparging used for aerating the culture in the fermenter increases air flow through the culture and can accelerate vaporization of limonene, thus leading to low titers; extensive reconfiguration will be required for alternative setups to trap limonene produced in the fermenter. Therefore, further optimization is required to overcome these hurdles.

### An investigation into the potential use of WCO as the carbon source for limonene production using the engineered *Y. lipolytica* strains

**Fig. 7:**
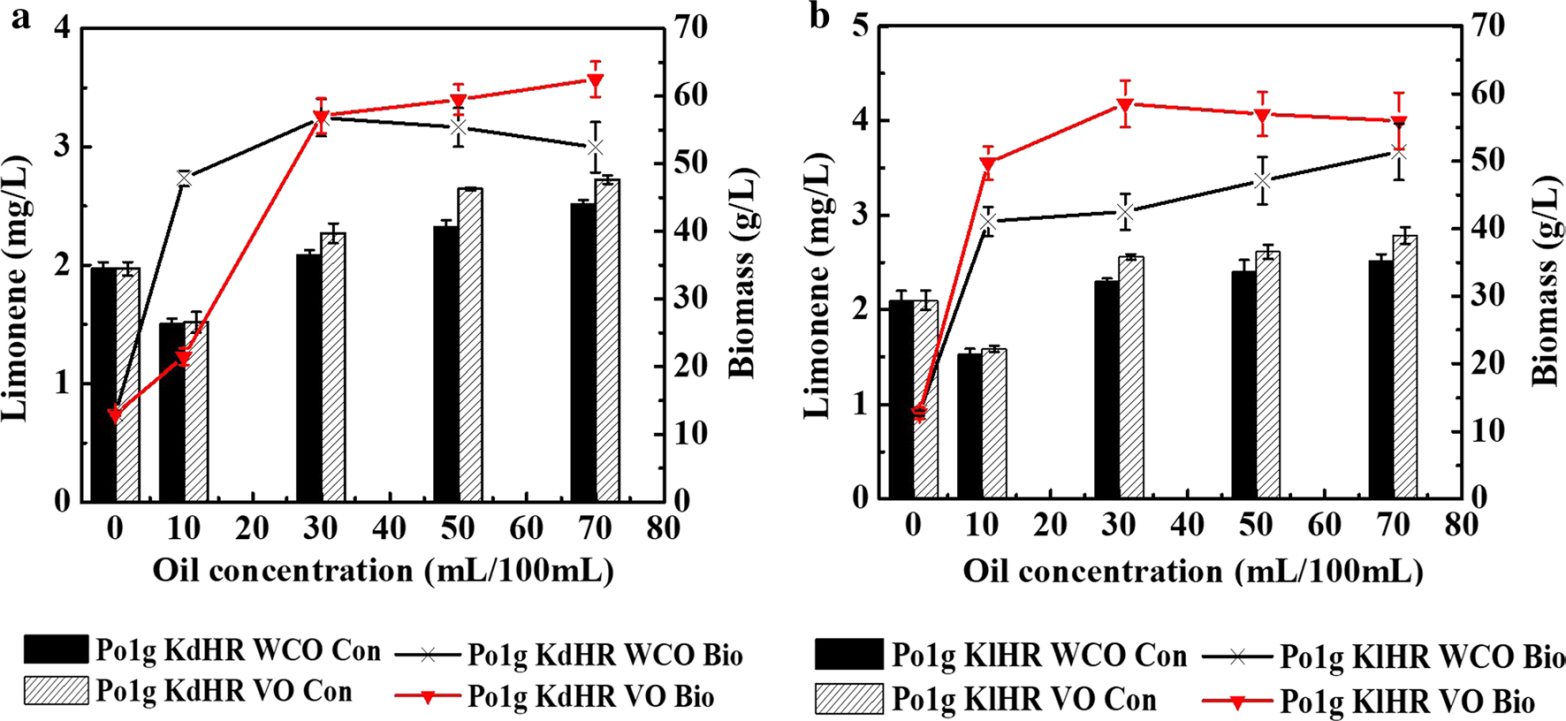
Effect of WCO on d-limonene or l-limonene accumulation in Po1g KdHR or Po1g KlHR. **a** Effect of WCO on d-limonene accumulation of Po1g KdHR.  : d-limonene accumulation of Po1g KdHR from WCO;  : d-limonene accumulation of Po1g KdHR from vegetable oil;  : biomass accumulation of Po1g KdHR from WCO;  : biomass accumulation of Po1g KdHR from vegetable oil. **b** Effect of WCO on l-limonene accumulation of Po1g KlHR.  : l-limonene accumulation of Po1g KlHR from WCO;  : l-limonene accumulation of Po1g KlHR from vegetable oil;  : biomass accumulation of Po1g KlHR from WCO;  : biomass accumulation of Po1g KlHR from vegetable oil. The cultivation of Po1g KdHR or Po1g KlHR was performed at the optimum fermentation temperature of 20 °C, the optimum rotation speed of 250 rpm, the optimum initial OD_600_ of 2.0, the optimum pH of 5.74, the best *n*-dodecane volume 10% and the optimum Mg^2+^ concentration 0.2% of MgSO_4_·7H_2_O in the optimal 50 mL-final-volume of WCO medium in a 250-mL shake flask for 5 days with different concentrations of WCO (0%, 10%, 30%, 50%, and 70%) as carbon source instead of glucose. Commercial vegetable oil was also used as a control.  : d-limonene accumulation of Po1g KdHR from WCO;  : l-limonene accumulation of Po1g KlHR from vegetable oil;  : biomass accumulation of Po1g KdHR;  : biomass accumulation of Po1g KlHR. All values presented are the mean of three biological replicates ± standard deviation

WCO mainly refers to frying oil used at high temperatures, edible fat mixed in kitchen waste and oily waste water directly discharged into the sewer. And annual global production of WCO exceeds 29 million tons [62]. Especially, China is the biggest producer of WCO in the world, with 4.5 million tons of WCO produced each year. If this waste is improperly handled, it will pollute the cities, as well as flow back to the dining tables in case of weak government supervisions, profit-driven practices and lack of rapid testing methods. According to the statistics, China’s WCO flowing back to the dining table amounts to 2.0–3.0 million tons, which poses a great health hazard to consumers [63]. Therefore, disposal and reutilization of WCO are long-term alarming problems in China. Disposal of WCO by appropriate management is thus vital [64, 65]. Traditionally, WCO has been used for diesel engine fuel production, soap fabrication, as additives in domestic animal feedstock or as an inexpensive cleaning agent [26]. However, most recent research contributions on bioremediation of WCO has focused on the utilization of WCO as raw and economical feedstock for production of different valuable products. These strategies simultaneously degraded WCO and obtained high value-added products, which would be more attractive methods for disposal of WCO from both economic and environmental protection perspectives [27, 66–70]. *Y. lipolytica* is capable of growing on various inexpensive carbon sources including alkanes, alkenes, fats, alcohols and organic acids. Owing to this unique ability of *Y. lipolytica*, we propose to investigate the potential use of WCO as the carbon source for limonene production from the engineered limonene-producing *Y. lipolytica* strains. To this end, Po1g KdHR and Po1g KlHR were cultured under different concentrations of WCO (0%, 10%, 30%, 50%, and 70%) as carbon source instead of glucose. Commercial vegetable oil was also used as a control (information on fatty acid composition of the commercial vegetable oil and waste cooking oil can be found in Additional file 1: Table S1). The cells were cultivated in 250-mL shake flasks for 5 days using the optimum fermentation parameters determined afore, i.e., temperature of 20 °C, rotation speed of 250 rpm and 0.2% of MgSO_4_·7H_2_O. The results showed d-limonene and l-limonene were successfully produced using WCO as sole carbon source, with the highest respective titers of 2.514 mg/L (Fig. 7a) and 2.723 mg/L (Fig. 7b) achieved with 70%. These represent approximately 11% of d-limonene and 16% of l-limonene increase in titers as compared to those obtained using glucose as carbon source and over 20-fold improvement compared with the initial titers of 0.124 mg/L d-limonene and 0.126 mg/L l-limonene before strain and fermentation optimization. Cell growth on WCO and vegetable oil was also monitored by determining the OD_600_ of Po1g KdHR and Po1g KlHR. With the same number of carbon units, the OD_600_ achieved by feeding 1.18% (w/v) WCO as carbon source was significantly higher than that with 2% glucose (Additional file 1: Fig. S3), demonstrating that our engineered limonene-producing *Y. lipolytica* strains can grow very efficiently and robustly on WCO as the sole carbon source, and the higher yield of limonene from WCO and vegetable oil may be attributed to the higher biomass (Fig. 7). Taken together, we have developed *Y. lipolytica* strains and optimized fermentation conditions that can potentially serve as a platform for sustainable and economical production of d-limonene and l-limonene from WCO.

## Conclusions

There is a great demand for limonene due to its wide range of industrial applications. Metabolic engineering of microorganisms has provided a platform for effective production of these valuable products. Here, we constructed a new pathway in *Y. lipolytica* using metabolic engineering tools to enzymatically convert the abundant acetyl-CoA pool in the oleaginous yeast host to d-limonene and l-limonene. Combining metabolic engineering strategies with optimization of fermentation conditions and fed-batch fermentation in shake flasks, final titers of 11.705 mg/L (0.443 mg/g) for d-limonene and 11.088 mg/L (0.385 mg/g) for l-limonene were achieved from glucose in this study. In addition, we also found that Mg^2+^ improved both the yields of d-limonene and l-limonene from the engineering yeast more than Mn^2+^. Importantly, we investigated the use of WCO to produce limonene and demonstrated that WCO is a superior substrate to glucose for producing limonene using the engineered limonene-producing *Y. lipolytica* strains. d-limonene and l-limonene were successfully produced at the respective titers of 2.514 mg/L and 2.723 mg/L with WCO as the carbon source. These are over 20-fold improvements compared to the initial titers of 0.124 mg/L d-limonene and 0.126 mg/L l-limonene before strain and fermentation optimization.

It is always beneficial that the biochemicals produced within cell factories are exported into the extracellular environment for collection in industrial applications. And the export of biochemicals has also been shown to successfully enhance production of target compounds. Although transport of hydrocarbons by passive diffusion or active transporters is known, the exact transport mechanism of limonene has not been reported [36, 37]. Therefore, we did not engineer the efflux/transportation of limonene in this study; much fundamental studies are required in future before this could be achieved. Furthermore, in this study, a dodecane overlay was used to extract limonene from the yeast culture because limonene could be trapped in a dodecane phase added to the medium during fermentation to enhance production level. Although this method is currently used widely for capturing volatile terpenes and other compounds produced by *Y. lipolytica* and different microbes, it may not be the best method for optimal harvest of limonene from this yeast, which exhibit alkane-binding behavior in large-scale production of limonene. In addition, the limonene production titers in this work are lower than that in previous reports (Table 1). The higher limonene productions were mostly achieved with *E. coli* but it is not the preferred host for industrial fermentation, particularly due to the susceptibility of *E. coli* to phage contamination [71]. Although higher limonene titer has been achieved in *Y. lipolytica* through extensive metabolic engineering [72], the unprecedented work reported here on optimizing fermentation conditions for limonene production has nevertheless provided valuable insights that will facilitate further efforts in the biosynthesis of this compound. In future, we will optimize the capture method for limonene from this yeast strain by evaluating the extraction efficiency of different methods [73], such as extraction with other solvents, solid-phase extraction and headspace trapping, along with the implementation of various engineering strategies to further improve the titer of limonene with our engineered *Y. lipolytica* strains.

In conclusion, we established that the oleaginous yeast strain *Y. lipolytica* is an attractive platform for providing a feasible and scalable route to limonene overproduction from WCO. Successful development of the *Y. lipolytica* platform to convert WCO into limonene will bring about a major breakthrough in the waste conversion and biochemical industries. However, the limonene downstream pathway was characterized in the present study. Given the accumulated knowledge about limonene downstream pathway and its regulation, it is believed that high productivity of limonene can be achieved by further strain engineering in our next experiments.

## Materials and methods

All chemicals, solvents, and media components were purchased and used without modification, Pml I, Kpn I, Spe I and Nru I were purchased from New England Biolabs (Beijing, China), ClonExpress^®^ II one step cloning kit, 2× Rapid taq master mix and 2× Phanta^®^ max master mix were purchased from Vazyme Biotech Co., Ltd. (Nanjing, China), tryptone and yeast extract were purchased from Thermo Scientific Oxoid Microbiology Products (Basingstoke, England), d-limonene and *n*-dodecane were purchased from Aladdin^®^ (Shanghai, China), l-limonene were purchased from Tokyo Chemical Industry (Shanghai, China), and salmon sperm DNA, plasmid extraction mini kit and DNA purification kit were purchased from Solarbio Life Sciences (Beijing, China). *Escherichia coli* DH5α was used for plasmid construction and amplification and *E. coli* strains were routinely cultured in LB medium supplemented with 100 μg/mL of ampicillin at 37 °C. *Y. lipolytica* Po1g KU70Δ was used as the base strain in this study. Routine cultivation of *Y. lipolytica* strains was carried out at 30 °C in YPD medium (yeast extract, 10 g/L; peptone, 20 g/L; glucose, 20 g/L). In experiments employing WCO as the carbon source, 2% glucose was removed and replaced with appropriate concentrations of WCO. WCO was collected from a local kitchen; vegetable oil was obtained commercially (Luhua brand). The growth medium was also supplemented with Tween-80 when WCO was used as the carbon source in WCO medium. All of the recombinant plasmids were constructed using the One Step Cloning Kit from Vazyme Biotech Co., Ltd (Nanjing, China). Plasmids used in this study are listed in Table 2 and strains are listed in Table 3. PCR primers used in this study were synthesized by Genewiz (Jiangsu, China) and are listed in Additional file 1: Table S2.

**Table 2:** Plasmids used in this study

Plasmid	Features	Reference
pYLEX1	*Y. lipolytica*‑integrative plasmid, P_hp4d_-T_XPR2_, LEU2	[78]
pYLdLS	P_hp4d_-dLS-T_XPR2_, LEU2	This study
pYLlLS	P_hp4d_-lLS-T_XPR2_, LEU2	This study
pYLA1	P_hp4d_-A1-T_XPR2_, LEU2	This study
pYLpA2	P_hp4d_-pA2-T_XPR2_, LEU2	This study
pYLA2	P_hp4d_-A2-T_XPR2_, LEU2	This study
pYLHS	P_hp4d_-HS-T_XPR2_, LEU2	This study
pYLHR	P_hp4d_-HR-T_XPR2_, LEU2	This study
pYLMK	P_hp4d_-MK-T_XPR2_, LEU2	This study
pYLPK	P_hp4d_-PK-T_XPR2_, LEU2	This study
pYLPD	P_hp4d_-PD-T_XPR2_, LEU2	This study
pYLIDI	P_hp4d_-IDI-T_XPR2_, LEU2	This study
pYLGS	P_hp4d_-GS-T_XPR2_, LEU2	This study
pYLFS	P_hp4d_-FS-T_XPR2_, LEU2	This study
pYLdA1	P_hp4d_-dLS-T_XPR2_, P_hp4d_-A1-T_XPR2_, LEU2	This study
PYLdA2	P_hp4d_-dLS-T_XPR2_, P_hp4d_-A2-T_XPR2_, LEU2	This study
pYLdHS	P_hp4d_-dLS-T_XPR2_, P_hp4d_-HS-T_XPR2_, LEU2	This study
pYLdHR	P_hp4d_-dLS-T_XPR2_, P_hp4d_-HR-T_XPR2_, LEU2	This study
pYLdMK	P_hp4d_-dLS-T_XPR2_, P_hp4d_-MK-T_XPR2_, LEU2	This study
pYLdPK	P_hp4d_-dLS-T_XPR2_, P_hp4d_-PK-T_XPR2_, LEU2	This study
pYLdPD	P_hp4d_-dLS-T_XPR2_, P_hp4d_-PD-T_XPR2_, LEU2	This study
pYLdIDI	P_hp4d_-dLS-T_XPR2_, P_hp4d_-IDI-T_XPR2_, LEU2	This study
pYLdGS	P_hp4d_-dLS-T_XPR2_, P_hp4d_-GS-T_XPR2_, LEU2	This study
pYLdFS	P_hp4d_-dLS-T_XPR2_, P_hp4d_-FS-T_XPR2_, LEU2	This study
PYLlA1	P_hp4d_-lLS-T_XPR2_, P_hp4d_-A1-T_XPR2_, LEU2	This study
PYLlA2	P_hp4d_-lLS-T_XPR2_, P_hp4d_-A2-T_XPR2_, LEU2	This study
pYLlHS	P_hp4d_-lLS-T_XPR2_, P_hp4d_-HS-T_XPR2_, LEU2	This study
pYLlHR	P_hp4d_-lLS-T_XPR2_, P_hp4d_-HR-T_XPR2_, LEU2	This study
pYLlMK	P_hp4d_-lLS-T_XPR2_, P_hp4d_-MK-T_XPR2_, LEU2	This study
pYLlPK	P_hp4d_-lLS-T_XPR2_, P_hp4d_-PK-T_XPR2_, LEU2	This study
pYLlPD	P_hp4d_-lLS-T_XPR2_, P_hp4d_-PD-T_XPR2_, LEU2	This study
pYLlIDI	P_hp4d_-lLS-T_XPR2_, P_hp4d_-IDI-T_XPR2_, LEU2	This study
pYLlGS	P_hp4d_-lLS-T_XPR2_, P_hp4d_-GS-T_XPR2_, LEU2	This study
pYLlFS	P_hp4d_-lLS-T_XPR2_, P_hp4d_-FS-T_XPR2_, LEU2	This study

**Table 3:** Strains used in this study

Strains	Genotype	Reference
Po1g KU70Δ	MatA, leu2-270, ura3-302::URA3, xpr2-332, axp-2, ku70-	[74]
Po1g KdLS	MatA, leu2-270, ura3-302::URA3, xpr2-332, axp-2, ku70-, dLS	This study
Po1g KlLS	MatA, leu2-270, ura3-302::URA3, xpr2-332, axp-2, ku70-, lLS	This study
Po1g KdA1	MatA, leu2-270, ura3-302::URA3, xpr2-332, axp-2, ku70-, dLS, ACOAAT1	This study
Po1g KdA2	MatA, leu2-270, ura3-302::URA3, xpr2-332, axp-2, ku70-, dLS, ACOAAT2	This study
Po1g KdHS	MatA, leu2-270, ura3-302::URA3, xpr2-332, axp-2, ku70-, dLS, HMGS	This study
Po1g KdHR	MatA, leu2-270, ura3-302::URA3, xpr2-332, axp-2, ku70-, dLS, HMGR	This study
Po1g KdMK	MatA, leu2-270, ura3-302::URA3, xpr2-332, axp-2, ku70-, dLS, MK	This study
Po1g KdPK	MatA, leu2-270, ura3-302::URA3, xpr2-332, axp-2, ku70-, dLS, PMK	This study
Po1g KdPD	MatA, leu2-270, ura3-302::URA3, xpr2-332, axp-2, ku70-, dLS, PMVADO	This study
Po1g KdIDI	MatA, leu2-270, ura3-302::URA3, xpr2-332, axp-2, ku70-, dLS, IPPDI	This study
Po1g KdGS	MatA, leu2-270, ura3-302::URA3, xpr2-332, axp-2, ku70-, dLS, GGPPS	This study
Po1g KdFS	MatA, leu2-270, ura3-302::URA3, xpr2-332, axp-2, ku70-, dLS, FPPS	This study
Po1g KlA1	MatA, leu2-270, ura3-302::URA3, xpr2-332, axp-2, ku70-, lLS, ACOAAT1	This study
Po1g KlA2	MatA, leu2-270, ura3-302::URA3, xpr2-332, axp-2, ku70-, lLS, ACOAAT2	This study
Po1g KlHS	MatA, leu2-270, ura3-302::URA3, xpr2-332, axp-2, ku70-, lLS, HMGS	This study
Po1g KlHR	MatA, leu2-270, ura3-302::URA3, xpr2-332, axp-2, ku70-, lLS, HMGR	This study
Po1g KlMK	MatA, leu2-270, ura3-302::URA3, xpr2-332, axp-2, ku70-, lLS, MK	This study
Po1g KlPK	MatA, leu2-270, ura3-302::URA3, xpr2-332, axp-2, ku70-, lLS, PMK	This study
Po1g KlPD	MatA, leu2-270, ura3-302::URA3, xpr2-332, axp-2, ku70-, lLS, PMVADO	This study
Po1g KlIDI	MatA, leu2-270, ura3-302::URA3, xpr2-332, axp-2, ku70-, lLS, IPPDI	This study
Po1g KlGS	MatA, leu2-270, ura3-302::URA3, xpr2-332, axp-2, ku70-, lLS, GGPPS	This study
Po1g KlFS	MatA, leu2-270, ura3-302::URA3, xpr2-332, axp-2, ku70-, lLS, FPPS	This study

### Plasmid construction

The d-limonene synthase gene (*dLS*, GenBank ID: AF514289) from *C. limon* and l-limonene synthase gene (*lLS*, GenBank ID: L13459) from *M. spicata* were synthesized and codon-optimized by Genewiz (Jiangsu, China). The sequences encoding the transit peptide within both genes were removed according to the previous literatures [27, 28]. The genes *dLS* and *lLS* were cloned into pYLEX1 (Additional file 1: Fig. S13) with primers dLS-F/dLS-R and lLS-F/lLS-R (Additional file 1: Table S2) to yield plasmid pYLdLS and pYLlLS (Additional file 1: Fig. S14), respectively. The intron-containing gene, pACOAAT2, was cloned into pYLEX1 with primers pACOAAT2-1-F/pACOAAT2-1-R and pACOAAT2-2-F/pACOAAT2-2-R to remove intron and yield plasmid pYLpA2, the gene ACOAAT2 was amplified from plasmid pYLpA2 and cloned into pYLEX1 with primers ACOAAT2-F/pACOAAT2-2-R to yield plasmid pYLA2. The genes ACOAAT1, HMGS, HMGR, MK, PMK, PMVADO, IPPDI, GGPPS, and FPPS were cloned into pYLEX1 with primers ACOAAT1-F/ACOAAT1-R, HMGS-F/HMGS-R, HMGR-F/HMGR-R, MK-F/MK-R, PMK-F/PMK-R, PMVADO-F/PMVADO-R, IPPDI-F/IPPDI-R, GGPPS-F/GGPPS-R, FPPS/FPPS-R to yield plasmid pYLA1, pYLHS, pYLHR, pYLMK, pYLPK, pYLPD, pYLIDI, pYLGS and pYLFS, respectively. The expression cassettes of ACOAAT1, ACOAAT2, HMGS, HMGR, MK, PMK, PMVADO, IPPDI, GGPPS and FPPS were cloned into pYLdLS and pYLlLS with primers BDH-F/BDH-R, BDH2-F/BDH2-R and FSBDH-F/BDH2-R to yield plasmid pYLdA1, pYLlA1, pYLdA2, pYLlA2, pYLdHS, pYLlHS, pYLdHR, pYLlHR, pYLdMK, pYLlMK, pYLdPK, pYLlPK, pYLdPD, pYLlPD, pYLdIDI, pYLlIDI, pYLdGS, pYLlGS, pYLdFS and pYLlFS (Additional file 1: Fig. S15), respectively. All plasmids were first linearized with Not I or Spe I and then transformed into competent cells of *Y. lipolytica* strains using the lithium acetate/single-stranded carrier DNA/polyethylene glycol method [74].

### Strain construction

Yeast colonies of *Y. lipolytica* Po1g KU70Δ were grown in 30 mL of fresh YPD medium for 24 h. Cells were pelleted and washed twice with 20 mL Tris–EDTA (TE) buffer (10 mM Tris, 1 mM EDTA, pH 7.5) and once with 0.1 M lithium acetate (pH 6.0). Then resuspend the cell with 3 mL of 0.1 M lithium acetate (pH 6.0), and incubate for 10 min at room temperature and aliquot 100 μL into sterile 1.5 mL tubes. The competent cells were then mixed by vortexing with 0.7 mL of 40% PEG-4000, 10 μL of denatured salmon sperm DNA and 10 μL of linearized recombination plasmids for 1 h. The transformation mixture and incubated at 39 °C for 1 h. Then add 1 mL YPD medium and recover for 2 h at 30 °C and 225 rpm. Following that, the transformation mixture was pelleted, resuspended in water and plated directly onto YNB plates. After selection, the following engineered *Y. lipolytica* strains were generated: Po1g KdLS, Po1g KlLS, Po1g KdA1, Po1g KlA1, Po1g KdA2, Po1g KlA2, Po1g KdHS, Po1g KlHS, Po1g KdHR, Po1g KlHR, Po1g KdMK, Po1g KlMK, Po1g KdPK, Po1g KlPK, Po1g KdPD, Po1g KlPD, Po1g KdIDI, Po1g KlIDI, Po1g KdGS, Po1g KlGS, Po1g KdFS and Po1g KlFS.

### Culturing the engineered *Y. lipolytica* strains for d-limonene and l-limonene production

To produce d-limonene and l-limonene, seed cultures were prepared by inoculating 5 mL of YPD medium in the 20-mL culture tubes with the engineered *Y. lipolytica* strains. The cells were grown at 30 °C for 24 h with agitation. Following that, 250-mL flasks containing 30 mL of YPD medium were inoculated to OD_600_ 0.1 with the seed cultures. All cultures were shaken at 200 rpm and 30 °C. To avoid loss of limonene during cultivation, 8% of *n*-dodecane overlay was added into the YPD medium prior to cultivation. Samples were then collected at day 5. To improve yields of d-limonene and l-limonene further, the effect of cultivation conditions was investigated, such as temperature, rotation speed, pH, the volume of culture medium, the volume of *n*-dodecane, initial OD_600_ and Mg^2+^ concentration. An example of optimization of temperature is given below, the cells were cultivated 200 rpm with an initial OD_600_ of 0.1 and 8% of *n*-dodecane in 30 mL of liquid YPD medium in a 250-mL shake flask for 5 days at various cultivation temperatures, i.e., 15, 20, 25 and 30 °C. Finally, different concentrations of WCO and vegetable oil (0%, 10%, 30%, 50%, and 70%) were added as carbon sources instead of glucose in YPD medium using the optimum fermentation parameters determined afore, and the dodecane layer was added into WCO medium before fermentation. At the end of fermentation, the mixture of the dodecane layer and WCO formed a one-layer system after centrifuging at 7500 rpm for 10 min. Therefore, the upper one-layer organic phase was used for analysis of limonene production by GC/MS.

### Fed-batch cultures

Fed-batch cultures were conducted in YPDM medium (yeast extract, 10 g/L; peptone, 20 g/L; glucose, 20 g/L; MgSO_4_·7H_2_O, 2 g/L). Overnight cultures of the engineered strains were diluted in 50 mL YPD to OD_600_ 2.0 in 250 mL shake flasks. Intermittent feeding with glucose was conducted at 24-h intervals, with 1 mL of 50% glucose being added at each feeding time-point. In the 5 L-bioreactor, the initial volume of YPDM medium was 2 L and continuous feeding was conducted, with 120 mL of 50% glucose fed at a constant rate over every 24-h cultivation period from 10 h to 72 h; 155 g glucose was fed in total. The pH value, temperature and oxygen saturation were fixed at 5.7 (regulated with 20% NaOH and 40% citric acid), 28 °C, and 20% (adjusted by stirring and aeration), respectively. AntiFoam B-396 (Guangdong Zhong Lian Bang Fine Chemical Co., Ltd, China) was used as the defoaming agent. Then, 5 mL of wet cell culture was harvested and washed twice with distilled water. The biomass was dried at 105 °C until the weight was stable.

### GC–MS analysis of d-limonene and l-limonene produced in the engineered *Y. lipolytica* strains

For the determination of d-limonene and l-limonene, all cultures were centrifuged at 7500 rpm for 10 min at each time of sampling. After fermentation, the volumes of *n*-dodecane phase were average 96% left compared to the initial volumes of *n*-dodecane. Specially, in the experiments using WCO as the sole carbon source, this volume of organic phase analyzed by GC–MS represents the total volume of WCO and *n*-dodecane. Then, 1 μL organic phase was then analyzed by GC–MS using an Agilent 7890A GC with an 5975C MSD equipped with a HP-5MS column (30 m × 0.25 mm × 0.25 μm, Agilent, Santa Clara, CA, USA). GC oven temperature was initially held at 60 °C for 2 min, and then ramped to 140 °C at a rate of 5 °C/min. It was then subsequently ramped at 10 °C/min to 280 °C and held for 5 min. The split ratio was 10:1. Helium was used as the carrier gas, with an inlet pressure of 13.8 psi. The injector was maintained at 280 °C and the ion source temperature was set to 230 °C. Final data analysis was achieved using Enhanced Data Analysis software (Agilent, Santa Clara, CA, USA).

## Supplementary information

**Additional file 1: Table S1.** Information on fatty acid composition of the commercial vegetable oil and waste cooking oil**. Table S2.** Primers used in PCR. **Figure S1.** Inhibitory effects of *d*‑limonene and l-limonene on *S. cerevisiae* AY15 and *Y. lipolytica* Po1g KU70Δ. **Figure S2.** GC–MS analysis of solvent overlay-extracted limonene from engineered strains cultures**. Figure S3.** Effect of different carbon sources on growth of *Y. lipolytica*. **Figure S4.** Effect of addition of glucose on biosynthesis of limonene. **Figure S5.** The OD_600_ values of different engineered *Y. lipolytica* strains cultured in YPD medium. **Figure S6.** Effect of temperature on volatilization of limonene. **Figure S7.** The pHs during fermentation of the engineered *Y. lipolytica* Po1g KdHR and Po1g KlHR strains in a 250-mL shake flask. **Figure S8.** The data of limonene batch-fermentation parameters in the 5 L-fermenter. **Figure S9.** Effect of Mg^2+^ on growth of *Y. lipolytica*. **Figure S10.** Effect of Mn^2+^ on d-limonene or l-limonene accumulation in Po1g KdHR or Po1g KlHR. **Figure S11.** Effect of concentration of peptone on the production of d-limonene and l-limonene. **Figure S12.** The data of limonene fed-batch fermentation parameters in the 5-L fermenter. **Figure S13.** Map of the plasmid pYLEX1. **Figure S14.** Map of the recombinant plasmid pYLdLS. **Figure S15.** Maps of the overexpression recombinant plasmid pYLA1 and pYLdA1. **Figure S16.** The GC–MS analysis of fatty acid composition in waste cooking oil and commercial vegetable oil.

## Data Availability

All relevant data generated or analyzed during this study were included in this published article.

## Abbreviations

GC/MS: Gas chromatography/mass spectrometry
OD_600_: Optical density at 600 nm
LB medium: 0.5% yeast extract, 1% tryptone and 1% NaCl
YPD medium: 1% yeast extract, 2% peptone and 2% glucose
WCO medium: 1% yeast extract, 2% peptone and appropriate concentration of WCO
YNB plate: 2% glucose, 0.67% yeast nitrogen base without amino acids and 2% agar
PCR: Polymerase chain reaction
DCW: Dry cell weight
