# Supplementary material for: Engineering the oleaginous yeast Yarrowia lipolytica to produce limonene from waste cooking oil
Source: Biotechnol Biofuels. 2019 Oct 8;12:241. doi: 10.1186/s13068-019-1580-y (PMC6781337; doi:10.1186/s13068-019-1580-y)
Supplement: Supplementary file 1 — Additional file 1: Table S1. Information on fatty acid composition of the commercial vegetable oil and waste cooking oil. Table S2. Primers used in PCR. Figure S1. Inhibitory effects of d‑limonene and l-limonene on S. cerevisiae AY15 and Y. lipolytica Po1g KU70Δ. Figure S2. GC–MS analysis of solvent overlay-extracted limonene from engineered strains cultures. Figure S3. Effect of different carbon sources on growth of Y. lipolytica. Figure S4. Effect of addition of glucose on biosynthesis of limonene. Figure S5. The OD600 values of different engineered Y. lipolytica strains cultured in YPD medium. Figure S6. Effect of temperature on volatilization of limonene. Figure S7. The pHs during fermentation of the engineered Y. lipolytica Po1g KdHR and Po1g KlHR strains in a 250-mL shake flask. Figure S8. The data of limonene batch-fermentation parameters in the 5 L-fermenter. Figure S9. Effect of Mg2+ on growth of Y. lipolytica. Figure S10. Effect of Mn2+ on d-limonene or l-limonene accumulation in Po1g KdHR or Po1g KlHR. Figure S11. Effect of concentration of peptone on the production of d-limonene and l-limonene. Figure S12. The data of limonene fed-batch fermentation parameters in the 5-L fermenter. Figure S13. Map of the plasmid pYLEX1. Figure S14. Map of the recombinant plasmid pYLdLS. Figure S15. Maps of the overexpression recombinant plasmid pYLA1 and pYLdA1. Figure S16. The GC–MS analysis of fatty acid composition in waste cooking oil and commercial vegetable oil. [file 13068_2019_1580_MOESM1_ESM.docx]

**ADDITIONAL INFORMATION**

**Engineering the oleaginous yeast *Yarrowia lipolytica* to produce limonene from waste cooking oil**

Yaru Pang, Yakun Zhao, Shenglong Li, Yu Zhao, Jian Li, Zhihui Hu, Cuiying Zhang, Dongguang Xiao, Aiqun Yu*

**Table S1. Information** **on fatty acid composition of the commercial vegetable oil and waste cooking oil**

| Fatty acid | Vegetable oil (Soybean oil, g/100g) | Waste cooking oil (g/100g) |
| --- | --- | --- |
| C8:0 | <0.000480 | 0.0406 |
| C14:0 | 0.0512 | 0.0520 |
| C15:0 | <0.000336 | 0.0021 |
| C16:0 | 9.3579 | 9.1128 |
| C16:1 | 0.0629 | 0.0592 |
| C17:0 | 0.0752 | 0.0704 |
| C17:1 | 0.0345 | 0.0286 |
| C18:0 | 3.8691 | 3.5801 |
| C18:1n9t | 0.0309 | 0.0953 |
| C18:1n9c | 20.1828 | 18.8678 |
| C18:2n6c | 55.0011 | 48.3580 |
| C20:0 | 0.3844 | 0.3847 |
| C18:3n6 | 0.9153 | 0.6781 |
| C20:1 | 0.5170 | 0.3990 |
| C18:3n3 | 6.9948 | 5.6221 |
| C21:0 | 0.0239 | 0.0386 |
| C20:2 | 0.0523 | 0.0492 |
| C22:0 | 0.5252 | 0.5505 |
| C23:0 | 0.0594 | 0.0616 |
| C20:5n3 | 0.4824 | 0.5014 |
| C24:1 | 0.0250 | 0.0364 |

The trademark of commercial vegetable oil was Luhua. Waste cooking oil was collected from the canteen of Tianjin University of Science and Technology, and the fatty acids of commercial vegetable oil and waste cooking oil were detected by Qingdao Sci-tech Innovation Quality Testing Co., Ltd.

**Table S2.** Primers used in PCR

| Primer | Sequence |
| --- | --- |
| dLS-F | AATGCGACGATCTGCCAACTAC |
| dLS-R | GG***GGTACC***TTAGTGGTGATGGTGATGATG |
| lLS-F | AATGGAGCGACGATCCGGCAATTATAATC |
| lLS-R | GG***GGTACC***TTAGTGGTGATGGTGATGATG |
| pYL-F | CCTCGATCCGGCATGCACTGATCACG |
| pYL-R | TAGGCAACAGCGTTGGGAGAGCCCTTGAGG |
| ACOAAT1-F | acaaccacacacatccacAATGCTACTCGACAGAAGAGACCTT |
| ACOAAT1-R | acaagttccgtagttggatccctaATGCGACTCACTCTGCCCC |
| pACOAAT2-1-F | CATacaaccacacacatccaGTTGAAGGAGACAGGTCAGAGTACG |
| pACOAAT2-1-R | gagtgcggCCTTCACGGCATGGGCTC |
| pACOAAT2-2-F | atgccgtgaaggCCGCACTCGCCAAGACCG |
| pACOAAT2-2-R | gggacaggccatggaggtaccCTAACACTTCTCAACAATGATAGAGG |
| ACOAAT2-F | acaaccacacacatccacAATGGAGCCCGTCTACATTGTTTC |
| HMGS-F | acaaccacacacatccacAATGTCGCAACCCCAGAACG |
| HMGS-R | gggacaggccatggaggtaccCTACTGCTTGATCTCGTACTTTCGTC |
| HMGR-F | acaaccacacacatccacAATGCTACAAGCAGCTATTGGAAA |
| HMGR-R | gggacaggccatggaggtaccCTATGACCGTATGCAAATATTCGAA |
| MK-F | acaaccacacacatccacAATGGACTACATCATTTCGGCG |
| MK-R | gggacaggccatggaggtaccCTAATGGGTCCAGGGACCGA |
| PMK-F | acaaccacacacatccacAATGCTACTTGAACCCCTTCTCG |
| PMK-R | gggacaggccatggaggtaccCTAATGACCACCTATTCGGCTCC |
| PMVADO-F | acaaccacacacatccacAATGATCCACCAGGCCTCCA |
| PMVADO-R | gggacaggccatggaggtaccCTACTTGCTGTTCTTCAGAGAACCA |
| IPPDI-F | acaaccacacacatccacAATGCTACTTGATCCACCGCC |
| IPPDI-R | gggacaggccatggaggtaccCTAATGACGACGTCTTACAGCGA |
| GGPPS-F | acaaccacacacatccacAATGTCACTGCGCATCCTCAA |
| GGPPS-R | gggacaggccatggaggtaccCTAATGGATTATAACAGCGCGGA |
| FPPS-F | acaaccacacacatccacAATGCTACTTCTGTCGCTTGTAAATC |
| FPPS-R | acaagttccgtagttggatccCTAATGTCCAAGGCGAAATTCGA |
| BDH-F | ccatccagcctcgcgtcgGTTAACTATCCTAGGGTGCATGCTGAG |
| BDH-R | acgtcttgctggcgttcgcgaTCATCGATGATAAGCTGTCAAACA |
| BDH2-F | ccatccagcctcgcgtcgGTTAACTATCCTAGGAGGCCGTTGAGCAC |
| BDH2-R | acgtcttgctggcgttcgATAAGCTGTCAAACATGAGAATTCG |
| FSBDH-F | ccatccagcctcgcgtcgACGCGTTATCCTAGGGTGCATGCTGAG |
| DJY-F | AATCGCCGTGACGATCAGC |
| DJY-R | CTGTCGCTTGCGGTATTCGG |
| ZHBDH-F | GCTTATCATCGATGATCGCGAAGTAGTAGGTTGAGGCCGTTG |
| ZHBDH-R | GCTACGTCTTGCTGGCGTTCGATGAGAATTCGGACACG |
| ZHYZ-F | CCAACAGCTTGCTCGCATC |
| ZHYZ-R | CCTCGCTCAAGCCTTCGTC |

The restriction sites are in bold, italic and underlined

**DNA alignment between codon-optimized and original sequence of *dLS* gene**


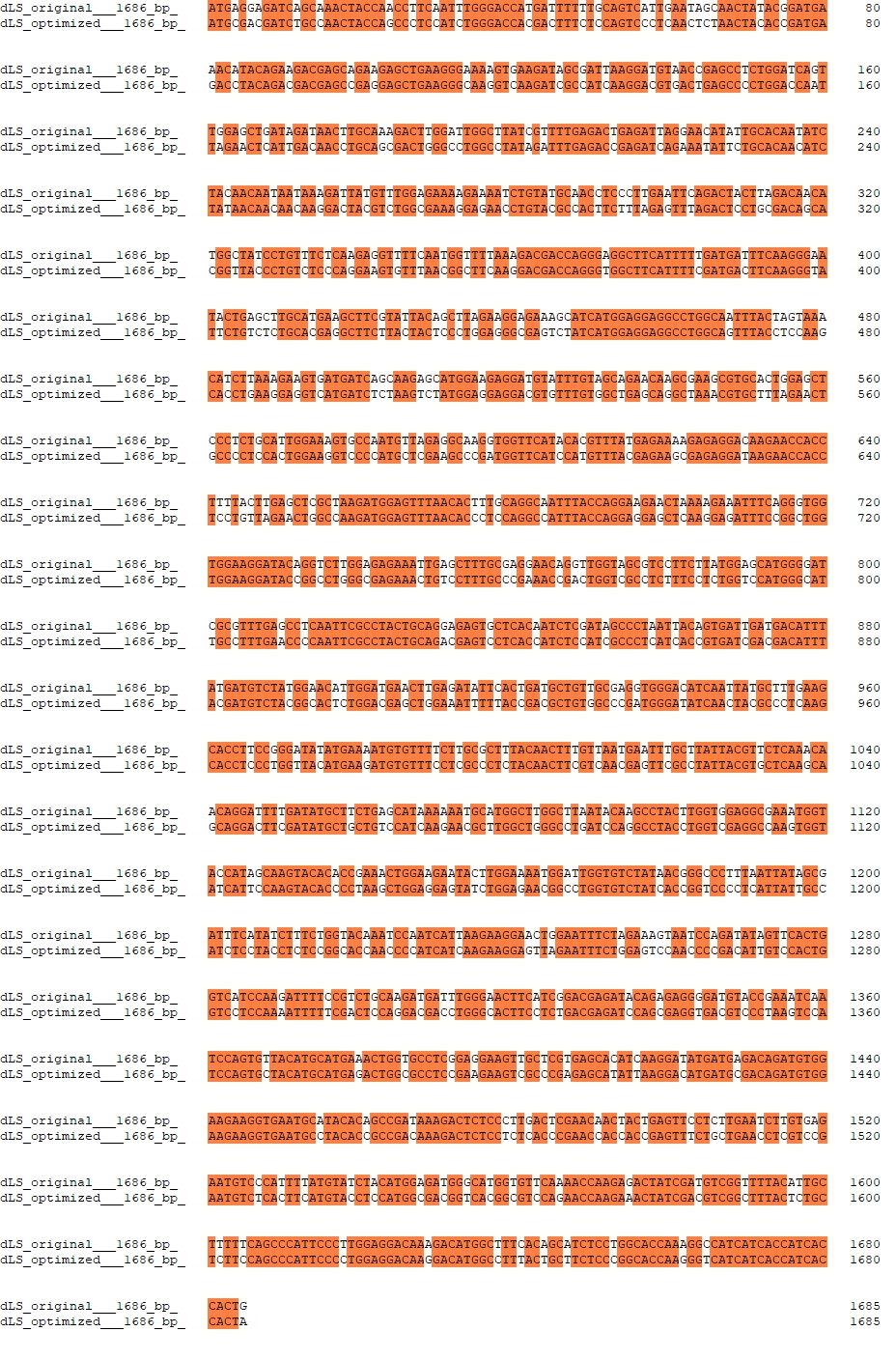


**DNA alignment between codon-optimized and original sequence of *lLS* gene**


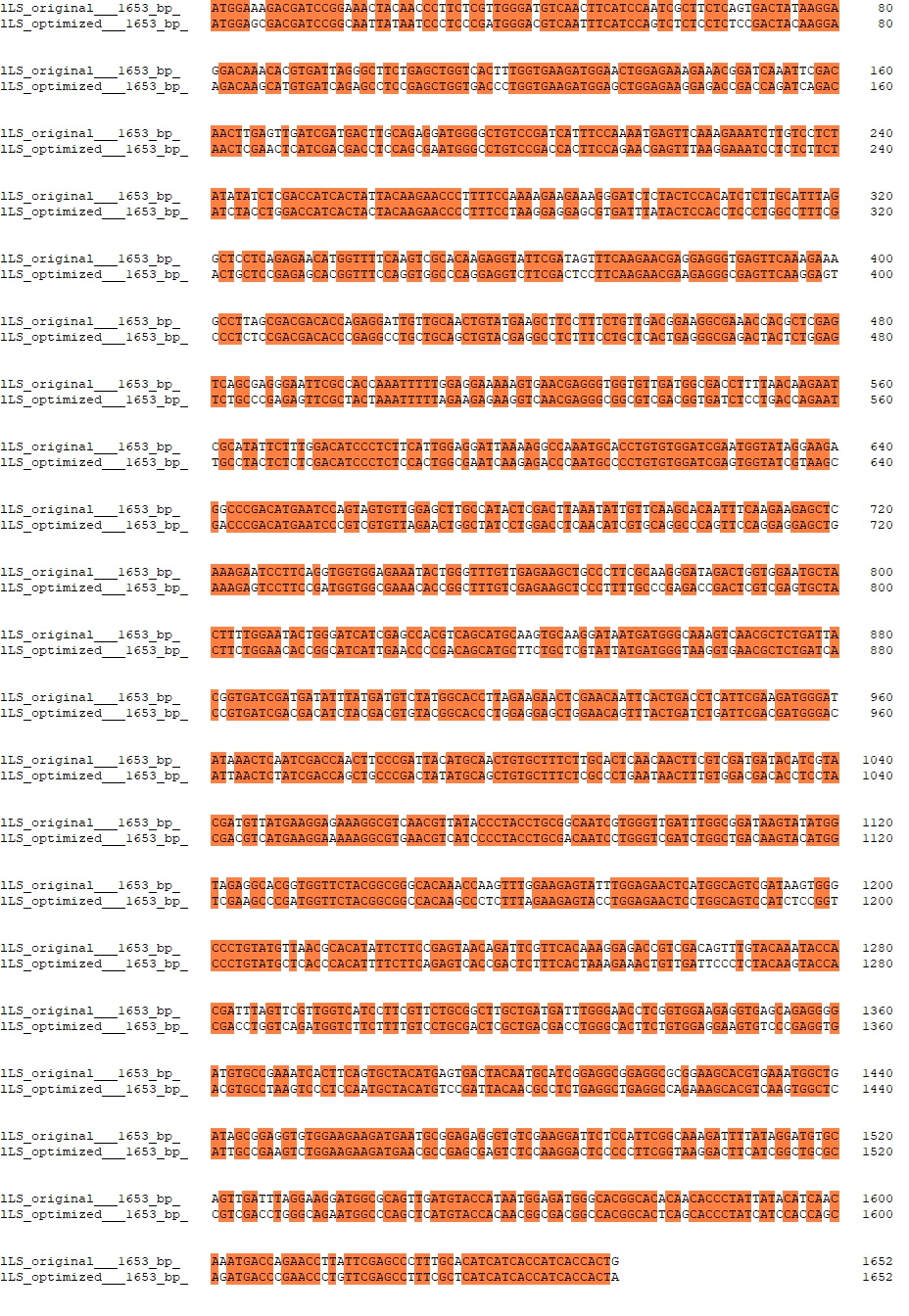


**Additional Methods**

**Preparation of *Y. lipolytica* Po1g** ***KU70*△ competent cell**

1. Inoculate a colony of *Y. lipolytica* Po1g *KU70*△ strain from a fresh YPD plate in 10 mL YPD medium (1% yeast extract, 2% peptone, 2% dextrose and 50 mM citrate buffer pH 4.0) in a 250 mL flask. Incubate with shaking at 225 rpm and 30°C for 20 hours.

2. Pellet the cells by centrifuging 5 minutes at 5, 000 g at 4℃.

3. Wash the cells with 20 mL TE buffer and pellet the cells similarly as Step 2.

4. Resuspend the cells in 1 mL of 0.1 M lithium acetate (pH 6.0, adjusted with acetic acid) and incubate for 10 minutes at room temperature.

5. Aliquot the competent cells (100 µL) into sterile 1.5 mL tubes. Proceed to the transformation steps below immediately, or add glycerol to a final concentration of 25% (v/v) and store at -80°C for long-term storage.

**Transformation of *Y. lipolytica* Po1g *KU70*△ cells**

1. Gently mix 10 µL of denatured salmon sperm DNA (10 mg/mL) and 1-5 µg of the linearized plasmid together with 100 µL of competent cells, and incubate at 30°C for 15 minutes.

2. Add 700 µL of 40% PEG-4000 (dissolved in 0.1 M lithium acetate pH 6.0), mix well and incubate at 30°C for 60 minutes with shaking (225 rpm).

3. Heat shock the transformation mixture at 39°C for 60 minutes.

4. Add 1 mL YPD medium and recover for 2 hours at 30°C and 225 rpm.

5. Centrifuge at 10,000 g for 1 minute, remove the supernatant and resuspend the pellet in 1 mL of TE buffer.

6. Repellet the cells and discard the supernatant again.

7. Resuspend the pellet in 100 µL of TE buffer and plate onto selective plates (leucine-deficient plates).

**Additional Figures**


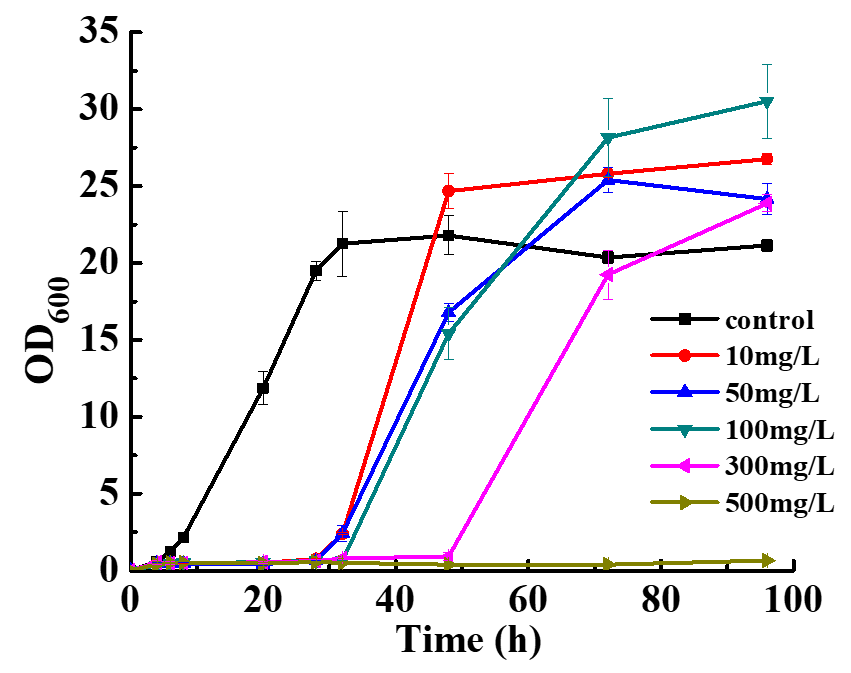


A


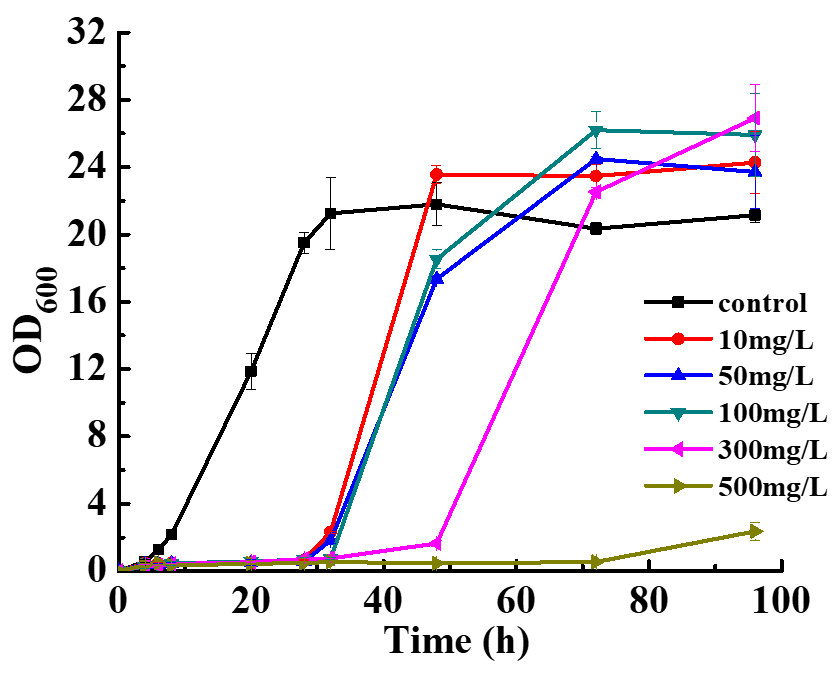


B


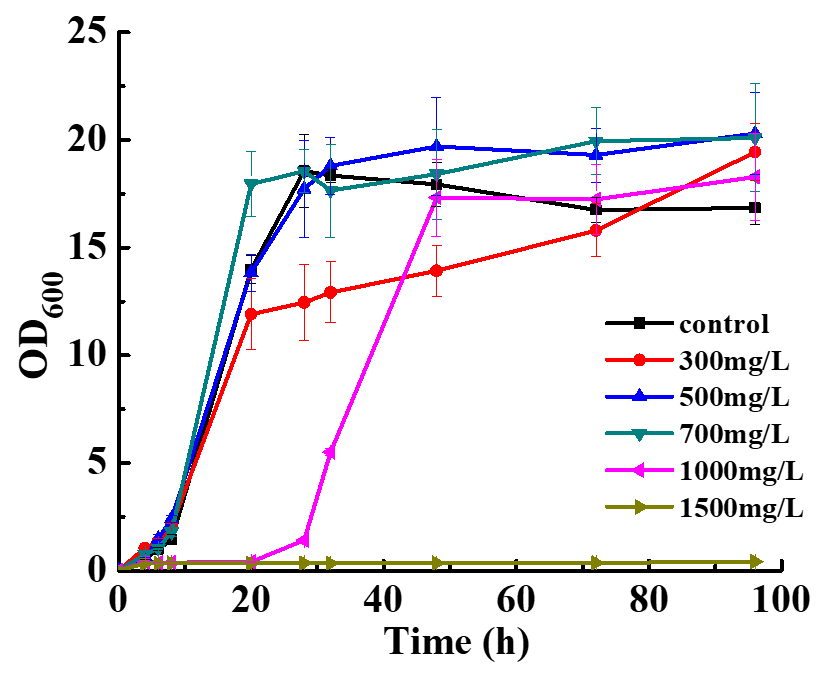


C


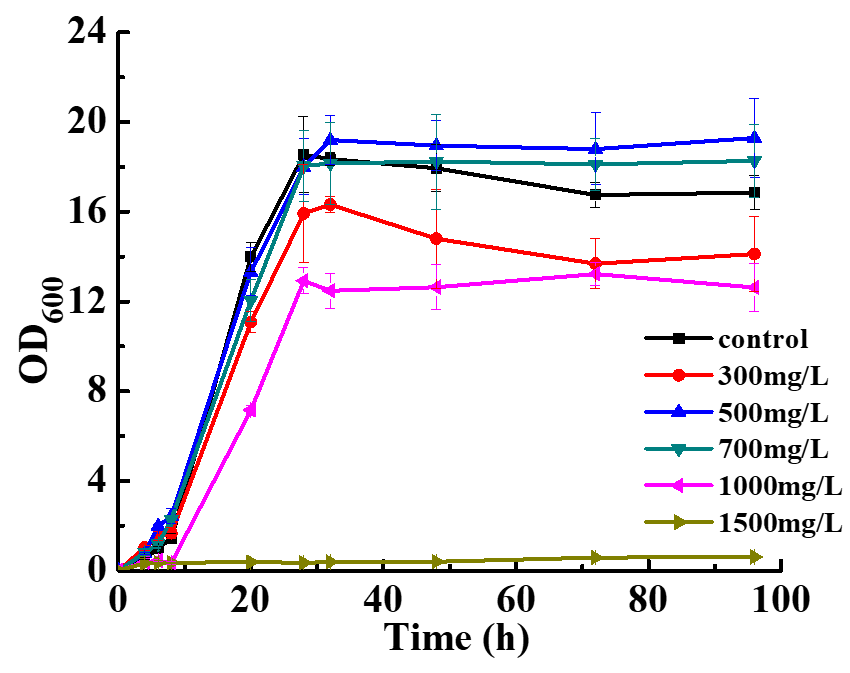


D

**Figure S1.** Inhibitory eﬀects of *d*‑limonene and *l*-limonene on *S. cerevisiae* AY15 and *Y. lipolytica* Po1g *KU70*△. (A) The inhibitory eﬀect of *d*‑limonene on *S. cerevisiae* AY15. (B) The inhibitory eﬀect of *l*-limonene on *S. cerevisiae* AY15. (C) The inhibitory eﬀect of *d*‑limonene on *Y. lipolytica* Po1g *KU70*△. (D) The inhibitory eﬀect of *l*-limonene on *Y. lipolytica* Po1g *KU70*△. A fixed volume of serially diluted *d*‑limonene and *l*-limonene in dodecane ranging from 10 to 1500 mg/L was added to YPD medium which contained 0.5 % Tween 80 (v v^‑1^), YPD medium containing 0.5 % Tween 80 (v v^‑1^) and dodecane was used as a negative control. The cultivation was performed at 30℃and 200 rpm in 30 mL of YPD medium. All values presented are the mean of three biological replicates ± standard deviation.


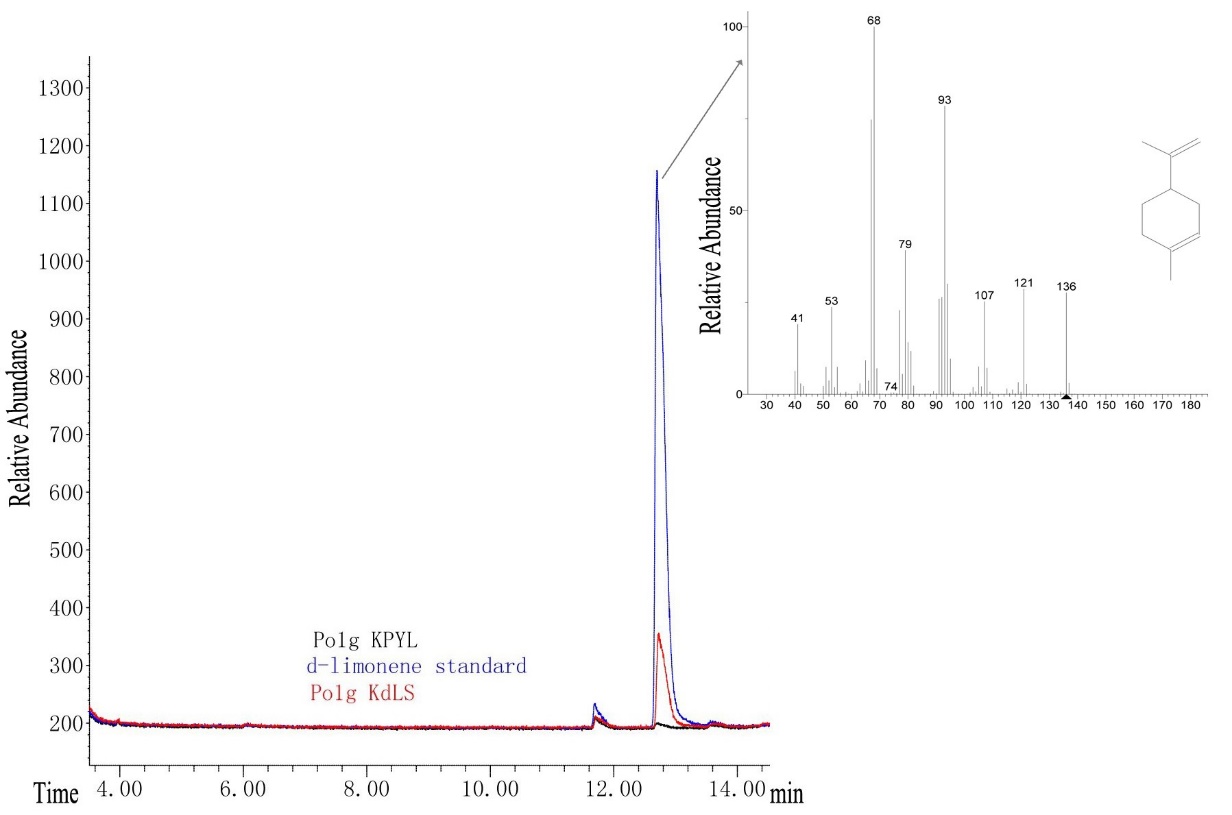


A


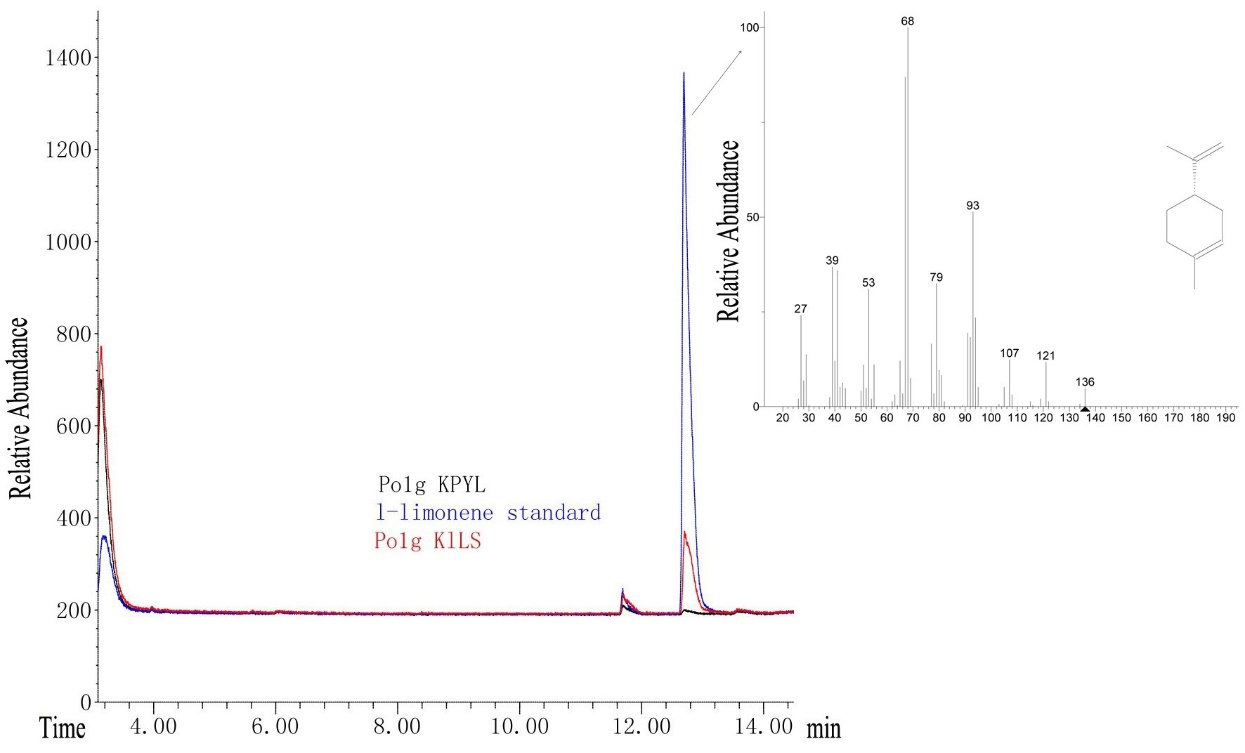


B

**Figure S2.** GC-MS analysis of solvent overlay-extracted limonene from engineered strains cultures. (A) *d*-limonene standard (10.027 mg/L) and *d*-limonene obtained in YPD medium of Po1g KPYL and Po1g KdLS; (B) *l*-limonene standard (11.225 mg/L) and *l*-limonene obtained in YPD medium of Po1g KPYL and Po1g KlLS. Po1g KPYL obtained by pYLEX1 integrating into Po1g △*KU70* was used as a negative control.


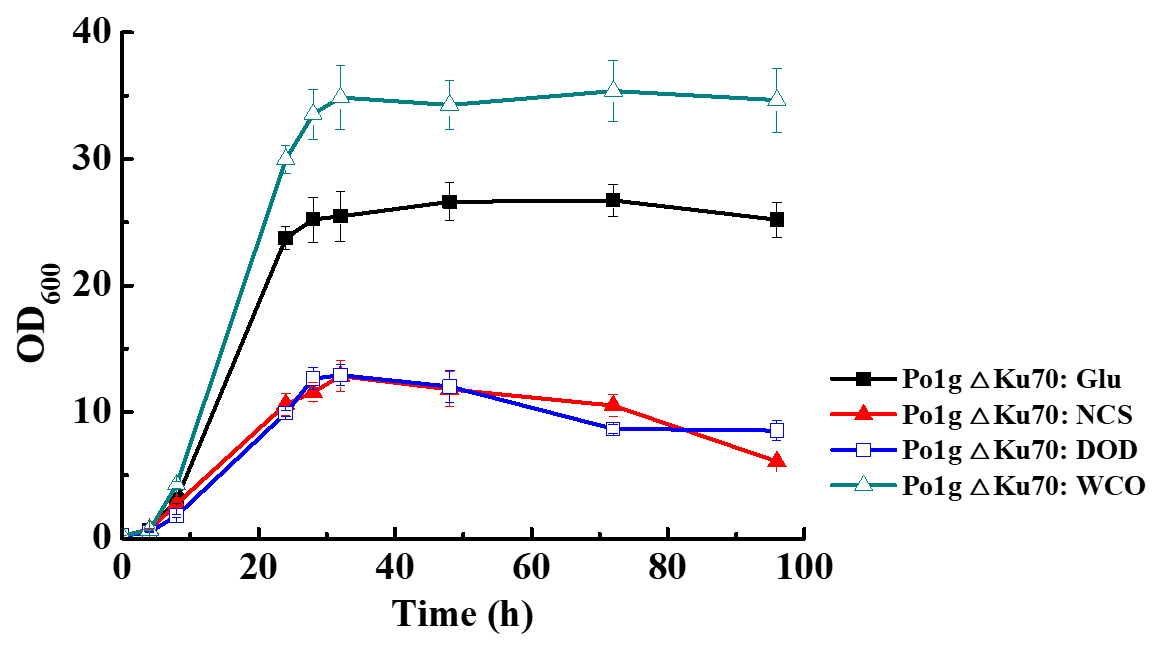


**Figure S3.** Effect of different carbon sources on growth of *Y. lipolytica*. Different carbon sources (glucose, waste cooking oil and dodecane) was added into the base medium, which included 1% yeast extract and 2% tryptone. OD_600_ was measured at 4 h, 8 h, 24 h, 28 h, 32 h, 48 h, 72 h and 96 h. Glu: 2% glucose was added into the base medium; NCS: there was no addition of any external carbon sources in the base medium; DOD: dodecane was added into the base medium; WCO: 1.18% (w/v) waste cooking oil as a carbon source which has an equal amount of carbon units to that of 2% (w/v) glucose was added into the base medium. All values presented are the mean of three biological replicates ± standard deviation.


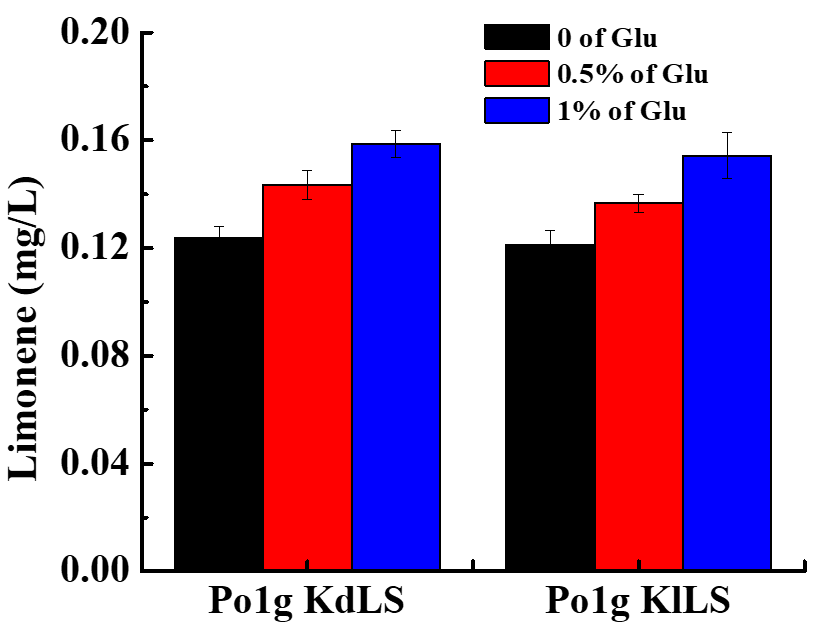


**Figure S4.** Effect of addition of glucose on biosynthesis of limonene. 0, 0.5% and 1% (w/v) of glucose was added into the culture medium of the engineered *Y. lipolytica* strains on day 3 and day 6, respectively. And the concentration of limonene was analyzed by GC-MS on day 9. All values presented are the mean of three biological replicates ± standard deviation.


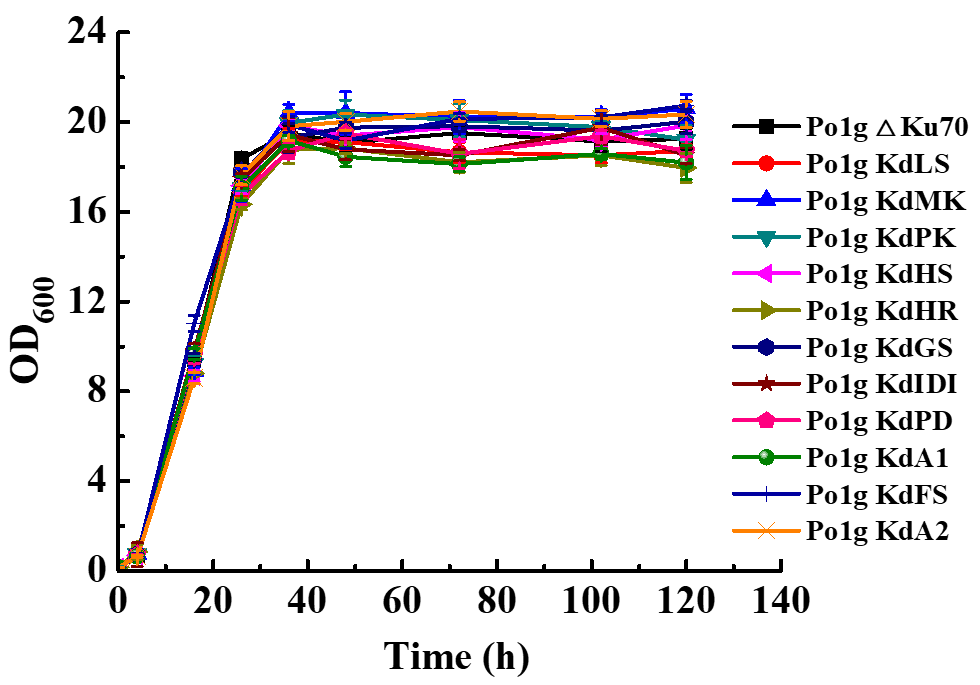

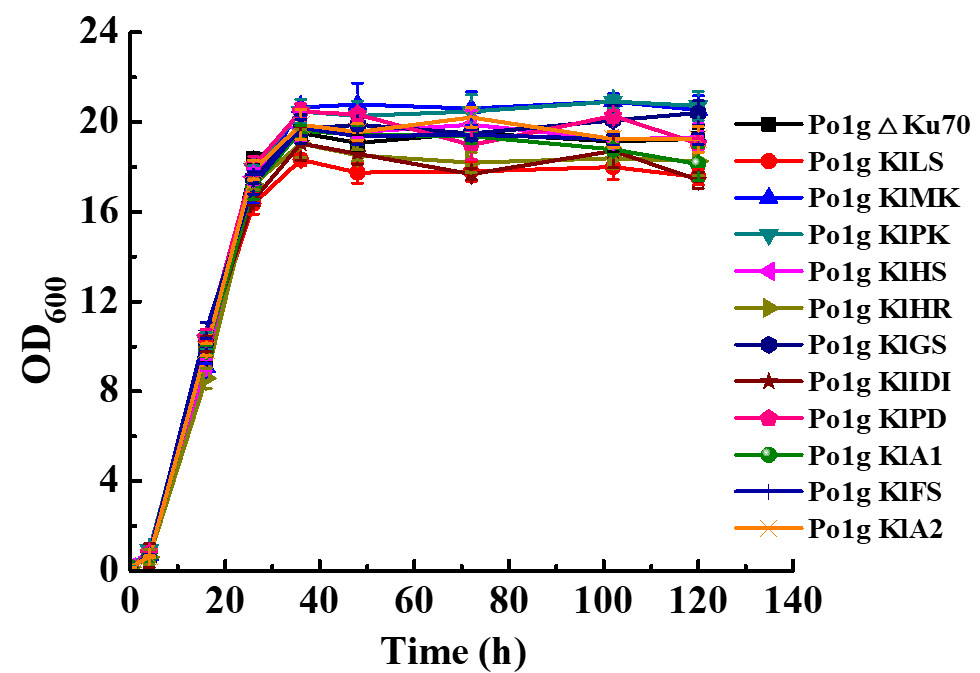


A B

**Figure S5.** The OD_600_ values of different engineered *Y. lipolytica* strains cultured in YPD medium. OD_600_ were measured at 0, 4, 16, 26, 32, 48, 72, 96 and 120 h, respectively. The cultivation was performed at 28℃ and 220 rpm. All values presented are the mean of three biological replicates ± standard deviation.


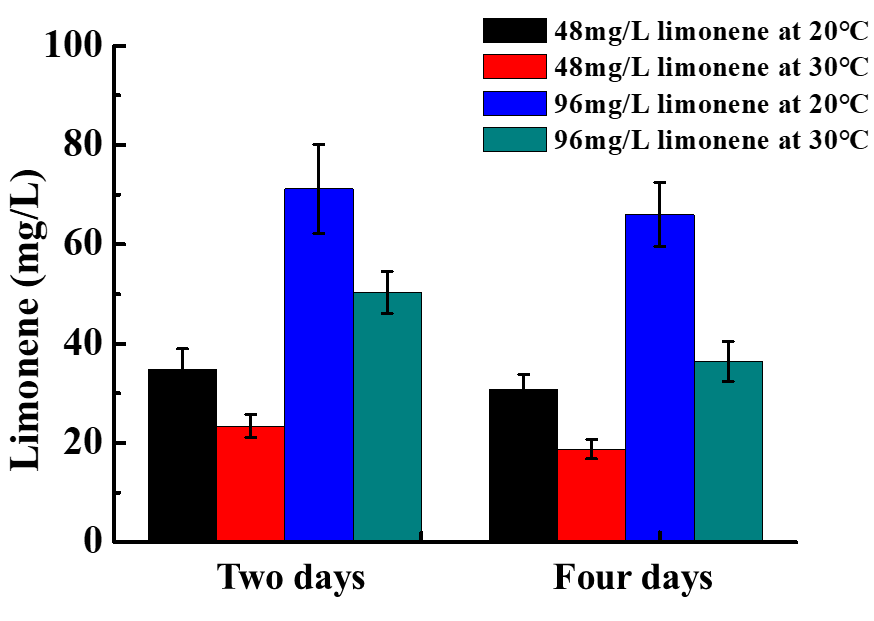


**Figure S6.** Effect of temperature on volatilization of limonene. The initial concentrations of limonene were 48 mg/L and 96 mg/L, respectively, which was dissolved in dodecane. Limonene was then added into the culture medium without *Y. lipolytica* cells. The concentration of limonene in the cell-free culture medium was analyzed by GC-MS after 2 days or 4 days of shaking at different temperatures (20℃ and 30℃). All values presented are the mean of three biological replicates ± standard deviation.


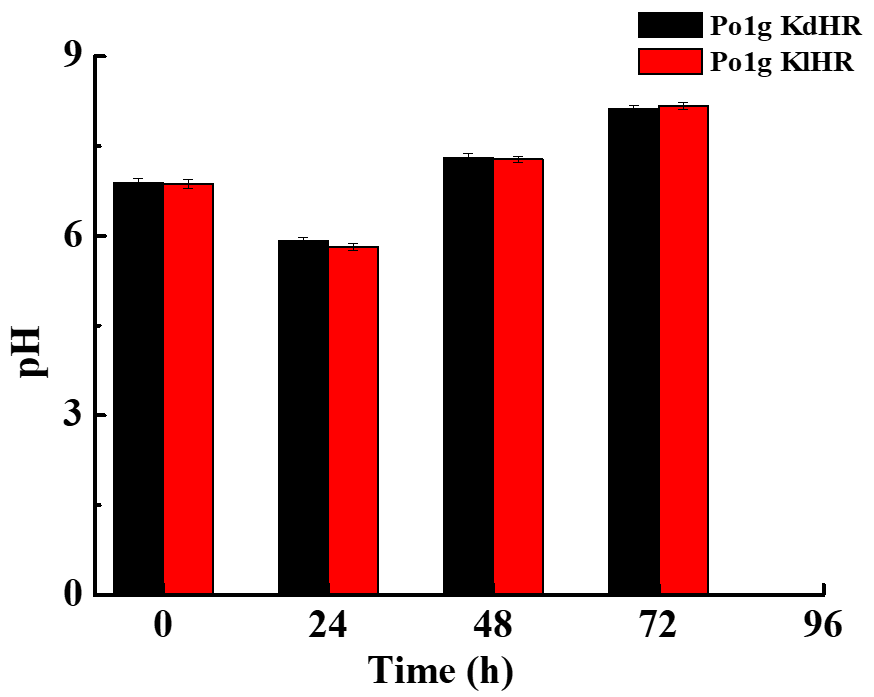


**Figure S7.** The pHs during fermentation of the engineered *Y. lipolytica* Po1g KdHR and Po1g KlHR strains in a 250-mL shake flask. The cultivation was carried out at 20℃, 250rpm, and pHs were measured at 0h 24h, 48h and 72h.


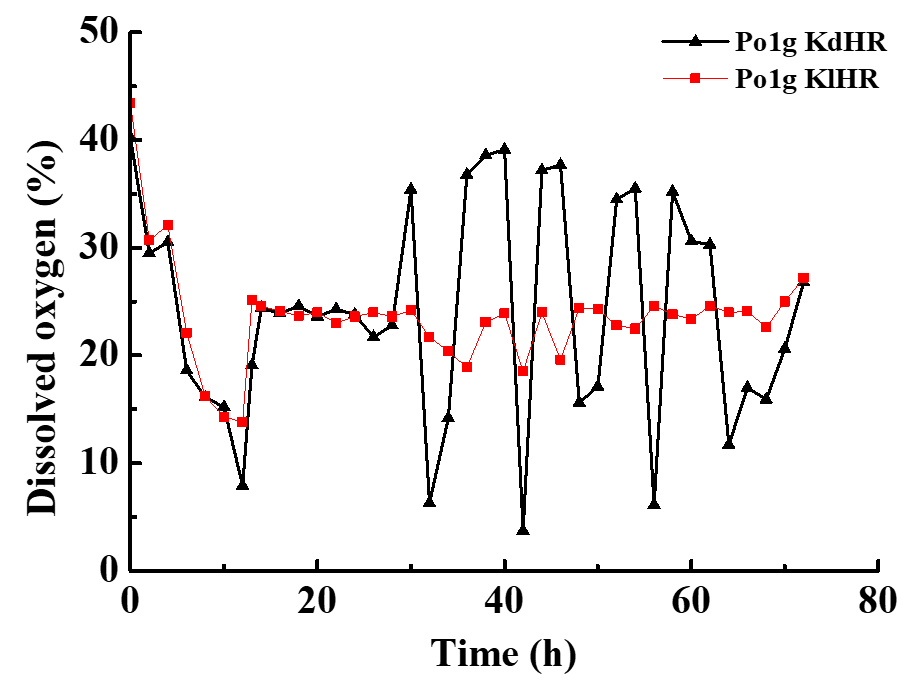


(a)


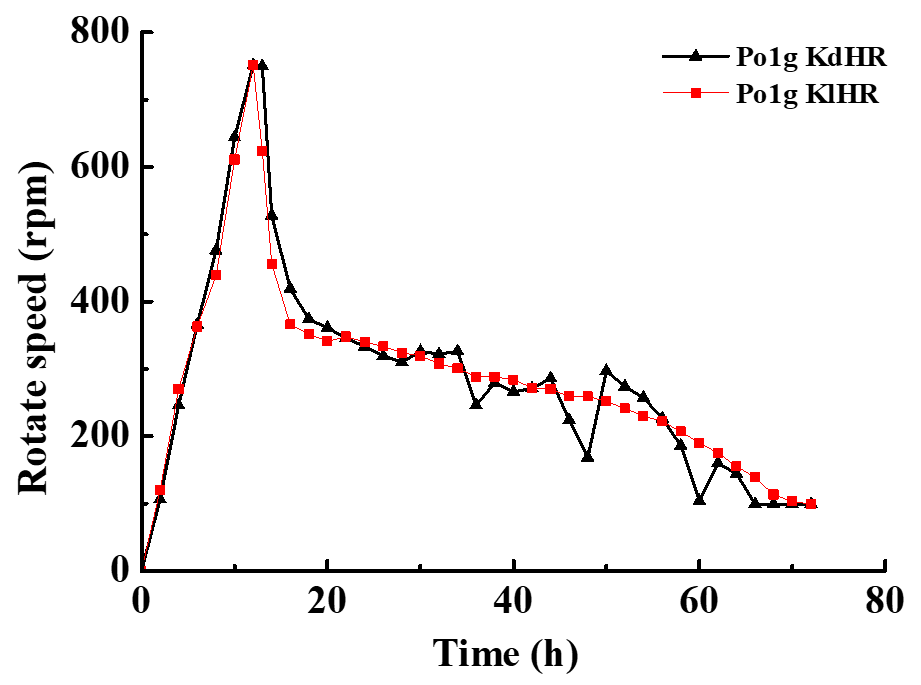


(b)


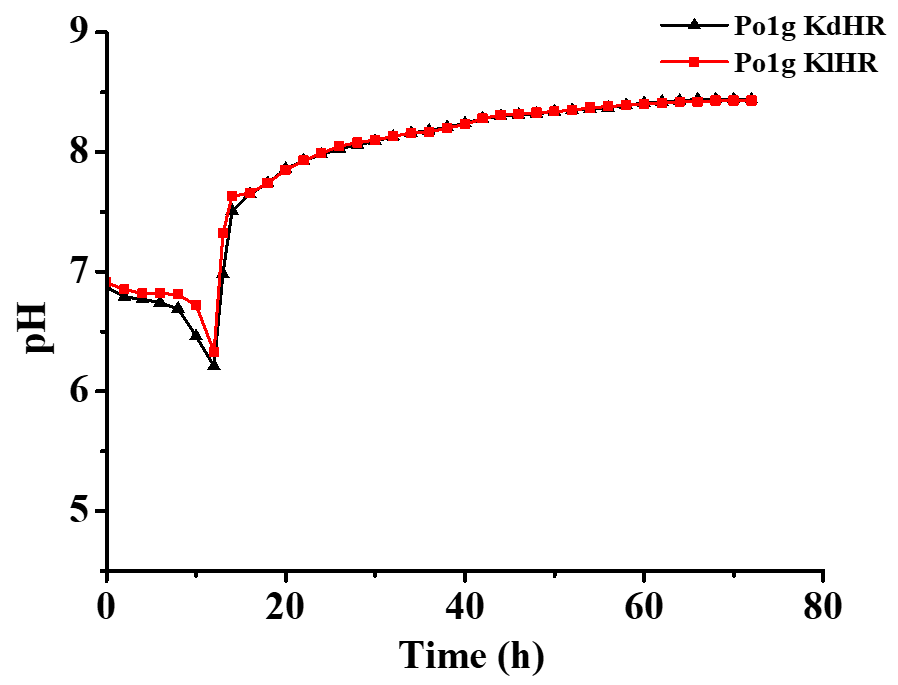


(c)

**Figure S8.** The data of limonene batch-fermentation parameters in the 5 L-fermenter. The initial volume of YPDM medium was 2 L, temperature and oxygen saturation were fixed at 28℃, and 20% (adjusted by stirring and aeration), respectively. (a) Change in dissolved oxygen of fermentation in the fermenter, which was set to 20%; (b) Change in rotate speed of fermentation in the fermenter, which was set to follow the level of dissolved oxygen; (c) Change in pH of fermentation medium in the fermenter.


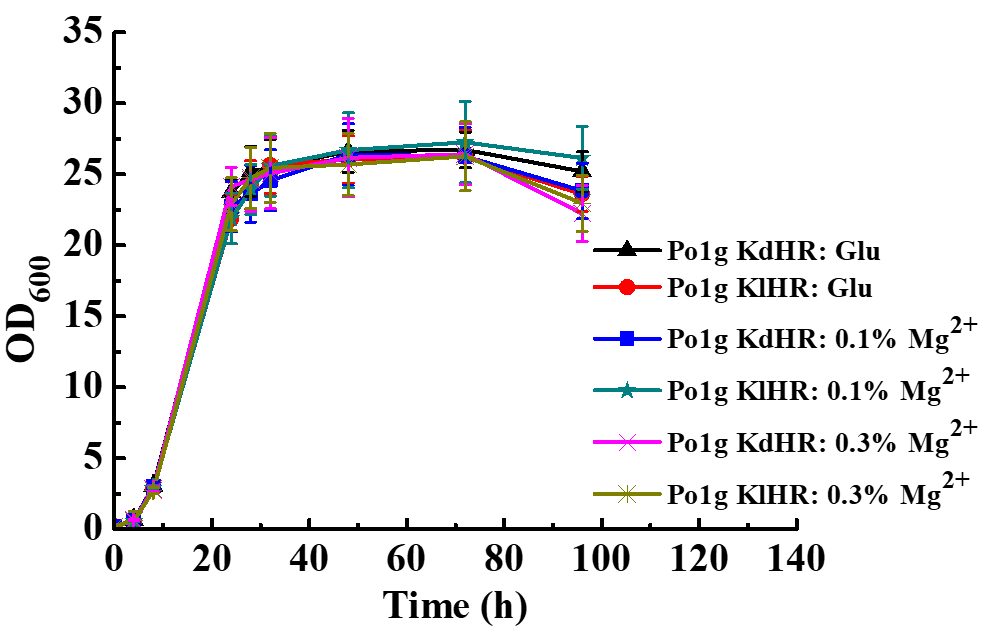


**Figure S9.** Effect of Mg^2+^ on growth of *Y. lipolytica*. Different concentration of MgSO_4_•7H_2_O was added into medium. OD_600_ was measured at 4 h, 8 h, 24 h, 28 h, 32 h, 48 h, 72 h and 96 h. All values presented are the mean of three biological replicates ± standard deviation.


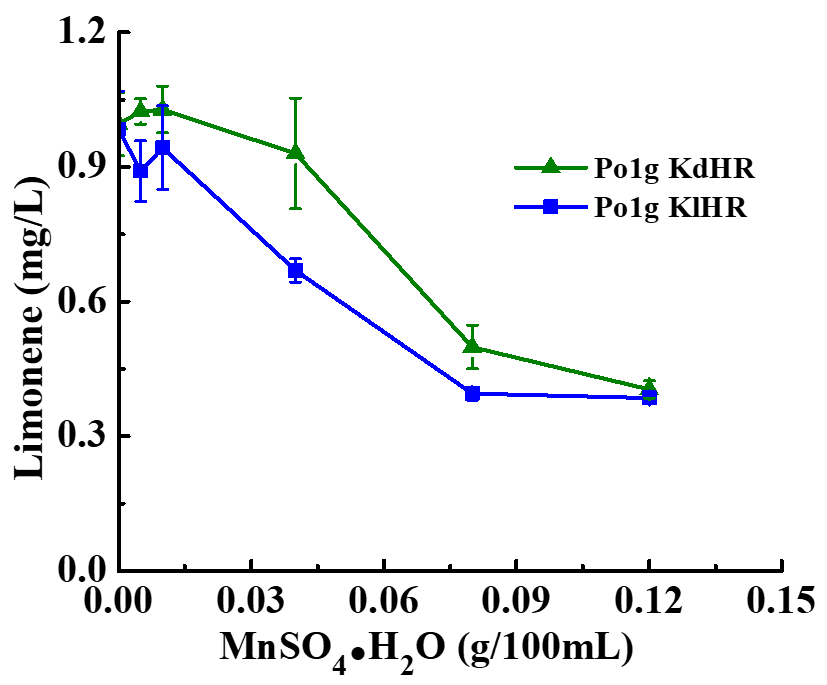


**Figure S10.** Effect of Mn^2+^ on *d*-limonene or *l*-limonene accumulation in Po1g KdHR or Po1g KlHR. The cultivation was performed at the optimum fermentation temperature of 20°C, the optimum rotation speed of 250 rpm, the optimum initial OD_600_ of 2.0, the optimum pH of 5.74 and the best n-dodecane volume 10% in the optimal 50 mL-final-volume of medium in a 250-mL shake flask for 5 days with different concentrations of MnSO_4_•7H_2_O (0, 0.005%, 0.01%, 0.04%, 0.08% and 0.12%). All values presented are the mean of three biological replicates ± standard deviation.


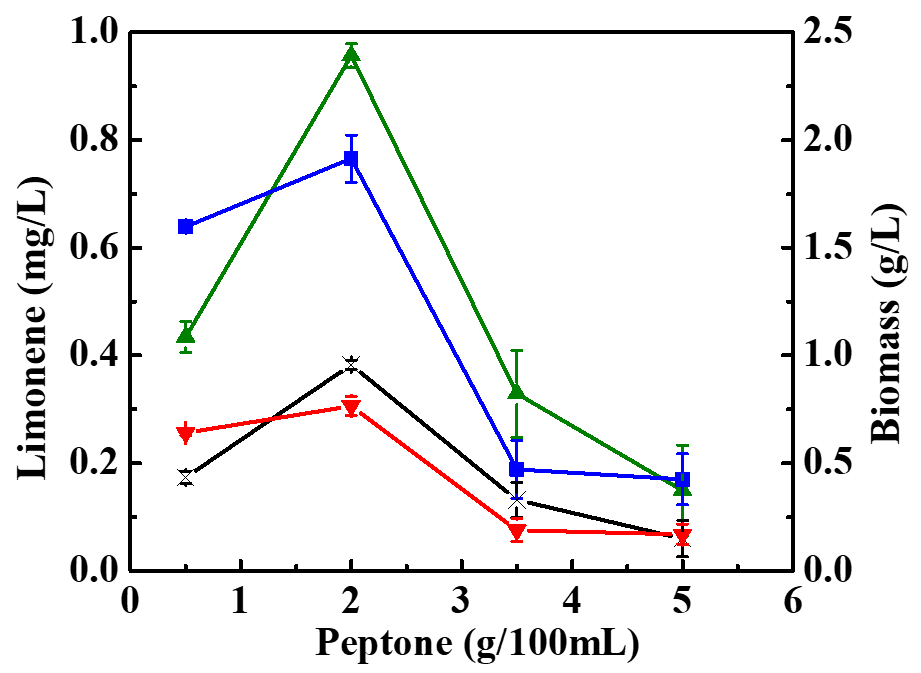


**Figure S11.** Effect of concentration of peptone on the production of *d*-limonene and *l*-limonene. Effect of peptone on *d*-limonene or *l*-limonene accumulation in Po1g KdHR or Po1g KlHR. The cultivation was performed at the optimum fermentation temperature of 20°C, the optimum rotation speed of 250 rpm, the optimum initial OD_600_ of 0.1, and the best n-dodecane volume 10% in the optimal 50 mL-final-volume of medium in a 250-mL shake flask for 5 days with different concentrations of peptone (0.5%, 2%, 3.5% and 5%). All values presented are the mean of three biological replicates ± standard deviation.


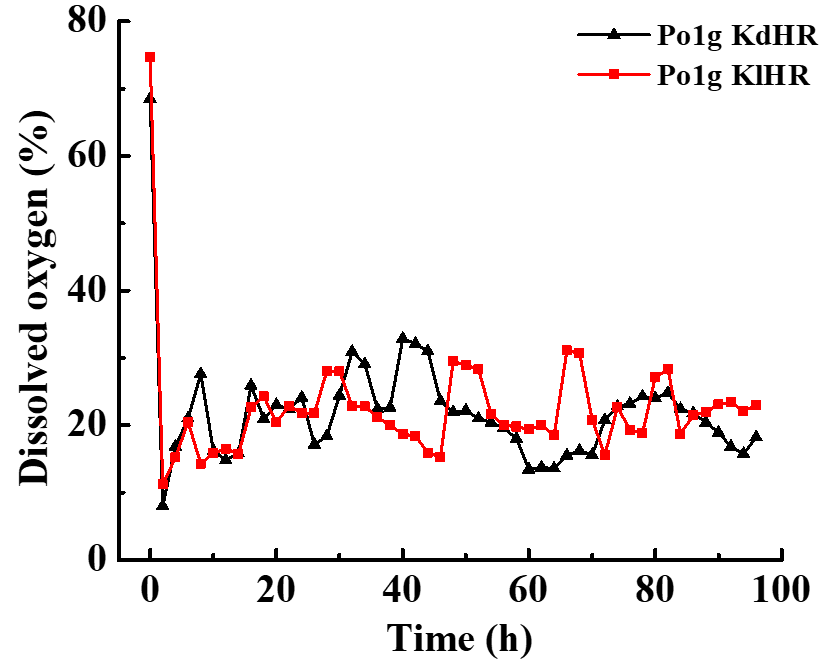


(a)


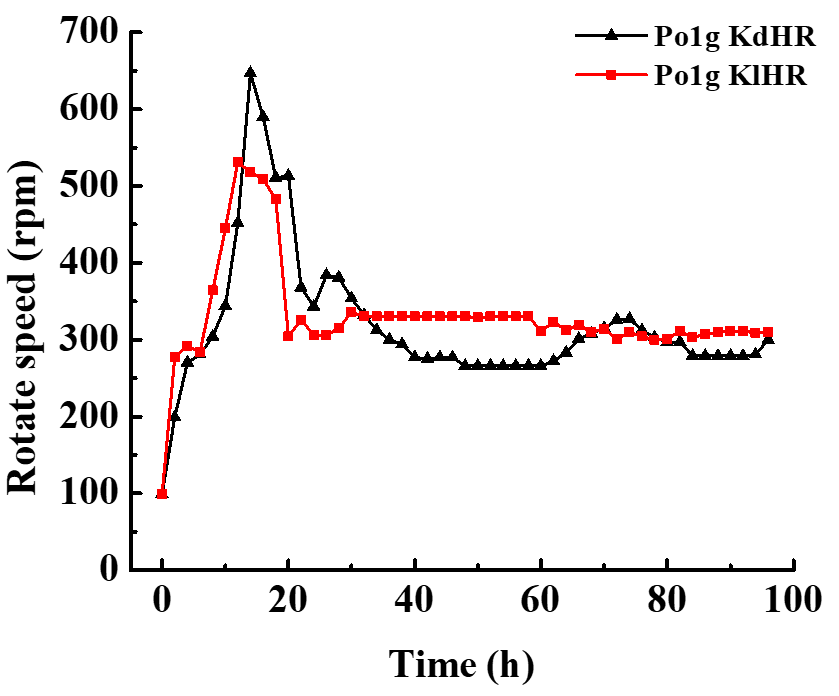


(b)


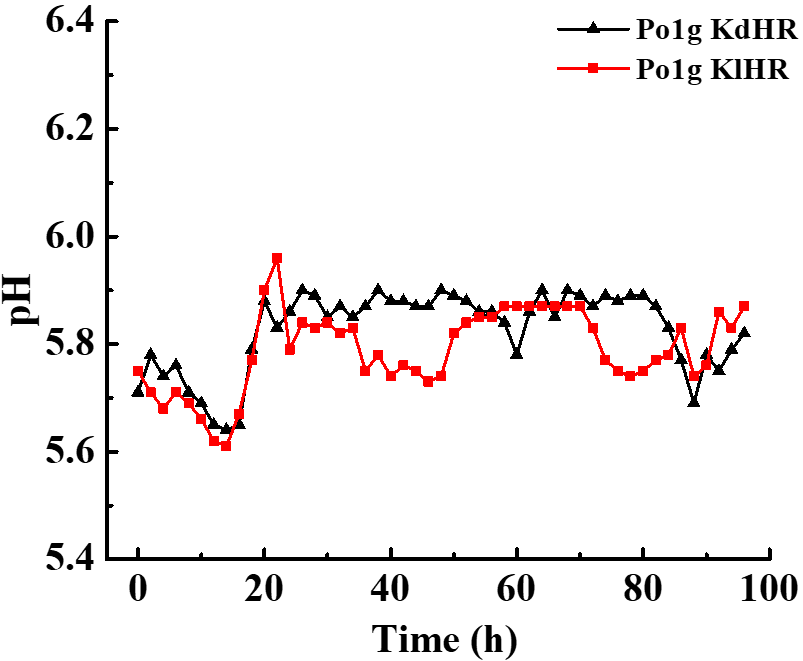


(c)

**Figure S12.** The data of limonene fed batch-fermentation parameters in the 5-L fermenter. (a) Change in dissolved oxygen of fermentation in the fermenter, which was set to 20%; (b) Change in rotate speed of fermentation in the fermenter, which was set to follow the level of dissolved oxygen; (c) Change in pH of fermentation medium in the fermenter.


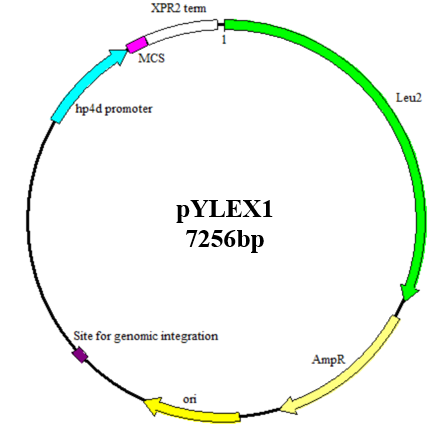


**Figure S13.** Map of the plasmid pYLEX1.


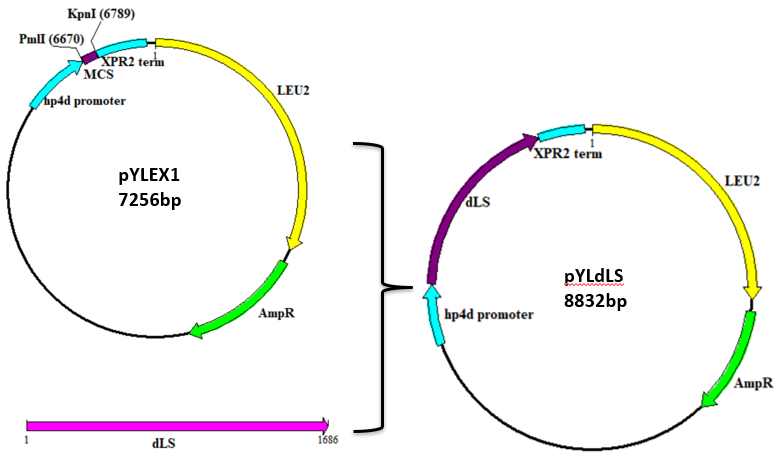


**Figure S14.** Map of the recombinant plasmid pYLdLS. The *dLS* gene were cloned into the Pml I/Kpn I site of pYLEX1 with a primer pair dLS-F/dLS-R to yield plasmid pYLdLS.


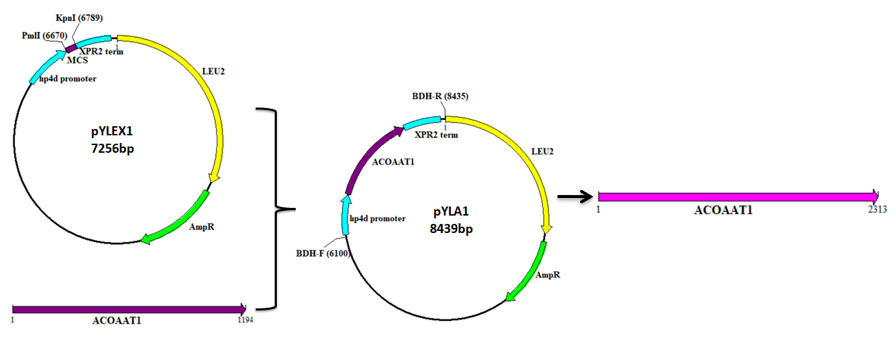


A


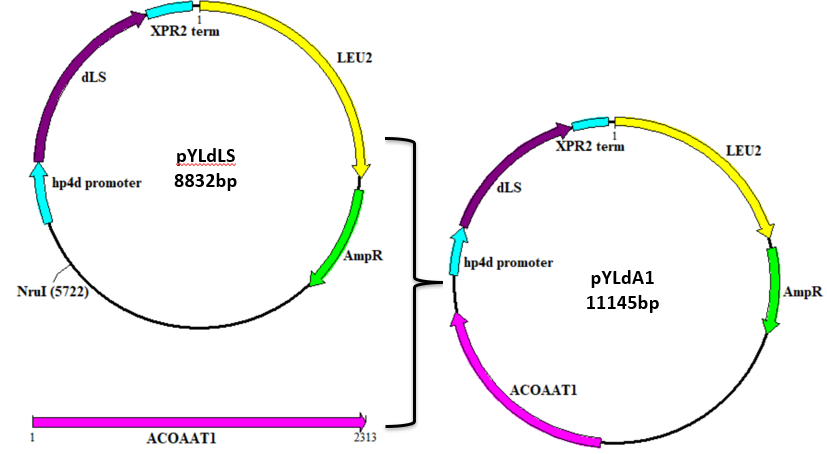


B

**Figure S15.** Maps of the overexpression recombinant plasmid pYLA1 and pYLdA1. ACOAAT1 were cloned into the Pml I/Kpn I site of pYLEX1 with primers ACOAAT1-F/ACOAAT1-R to yield plasmid pYLA1 (A). The expression cassette of ACOAAT1 were cloned into pYLdLS with primers BDH-F/BDH-R to yield plasmid pYLdA1 (B).


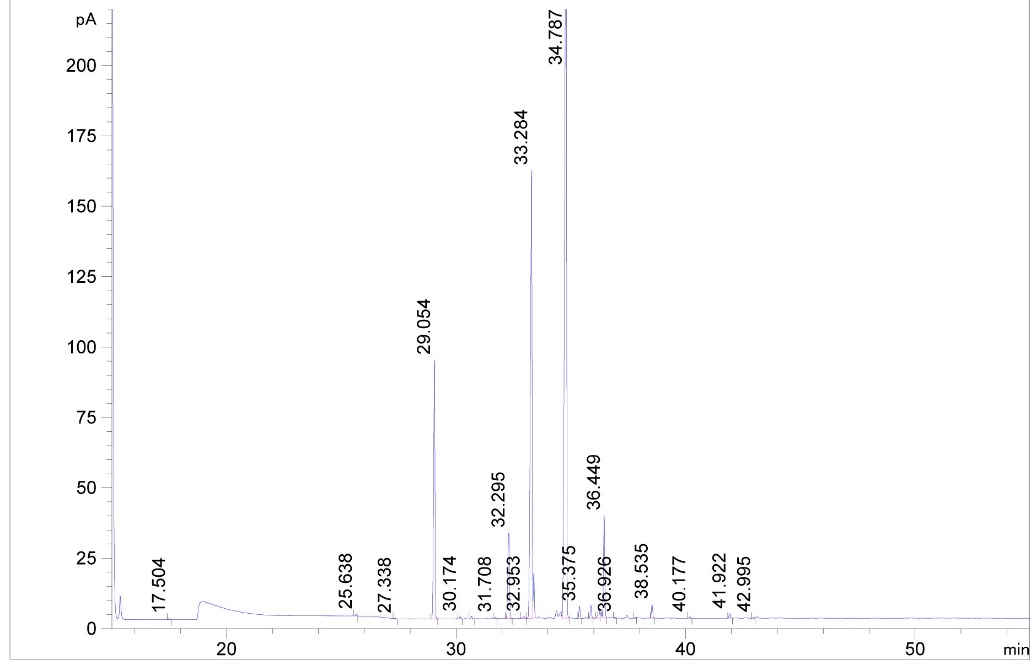


(a)


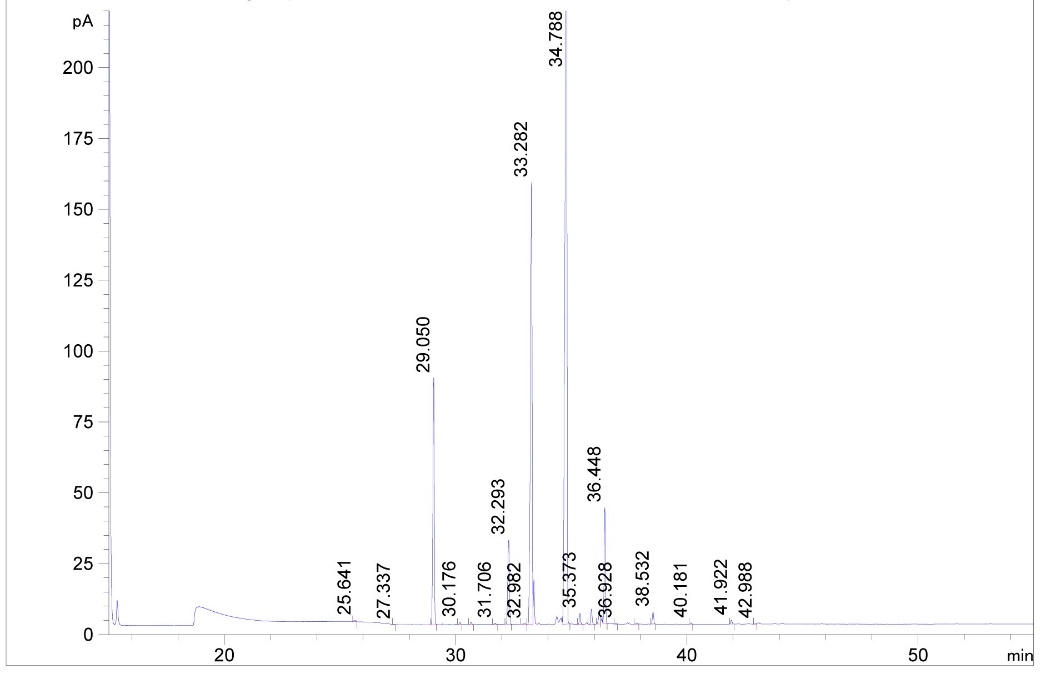


(b)

**Figure S16.** The GC-MS analysis of fatty acid composition in waste cooking oil and commercial vegetable oil. (A) The GC-MS analysis of fatty acid composition in waste cooking oil; (B) The GC-MS analysis of fatty acid composition in commercial vegetable oil.
